# A Systematic Review Characterizing On-Farm Sources of *Campylobacter* spp. for Broiler Chickens

**DOI:** 10.1371/journal.pone.0104905

**Published:** 2014-08-29

**Authors:** Agnes Agunos, Lisa Waddell, David Léger, Eduardo Taboada

**Affiliations:** 1 Laboratory for Foodborne Zoonoses, Public Health Agency of Canada, Guelph, Ontario, Canada; 2 Department of Population Medicine, University of Guelph, Guelph, Ontario, Canada; 3 Laboratory for Foodborne Zoonoses, Public Health Agency of Canada, Lethbridge, Alberta, Canada; Wageningen University and Research Centre, Netherlands

## Abstract

*Campylobacter* and antimicrobial-resistant *Campylobacter* are frequently isolated from broiler chickens worldwide. In Canada, campylobacteriosis is the third leading cause of enteric disease and the regional emergence of ciprofloxacin-resistant *Campylobacter* in broiler chickens has raised a public health concern. This study aimed to identify, critically appraise, and synthesize literature on sources of *Campylobacter* in broilers at the farm level using systematic review methodology. Literature searches were conducted in January 2012 and included electronic searches in four bibliographic databases. Relevant studies in French or English (n = 95) conducted worldwide in any year and all study designs were included. Risk of Bias and GRADE criteria endorsed by the Cochrane collaboration was used to assess the internal validity of the study and overall confidence in the meta-analysis. The categories for on-farm sources were: broiler breeders/vertical transfer (number of studies = 32), animals (n = 57), humans (n = 26), environment (n = 54), and water (n = 63). Only three studies examined the antimicrobial resistance profiles of *Campylobacter* from these on-farm sources. Subgroups of data by source and outcome were analyzed using random effect meta-analysis. The highest risk for contaminating a new flock appears to be a contaminated barn environment due to insufficient cleaning and disinfection, insufficient downtime, and the presence of an adjacent broiler flock. Effective biosecurity enhancements from physical barriers to restricting human movement on the farm are recommended for consideration to enhance local on-farm food safety programs. Improved sampling procedures and standardized laboratory testing are needed for comparability across studies. Knowledge gaps that should be addressed include farm-level drug use and antimicrobial resistance information, further evaluation of the potential for vertical transfer, and improved genotyping methods to strengthen our understanding of *Campylobacter* epidemiology in broilers at the farm-level. This systematic review emphasizes the importance of improved industry-level and on-farm risk management strategies to reduce pre-harvest *Campylobacter* in broilers.

## Introduction

Molecular epidemiologic studies have identified poultry as the most important source of human campylobacteriosis in industrialized countries [1]–[3]. However, *Campylobacter* in broilers has received little attention in comparison to other microbial hazards, e.g. *Salmonella* that have more severe sequelae in humans. In Canada, the current estimated annual incidence of campylobacteriosis is 447.23 cases per 100,000 in the population, making campylobacteriosis the third leading cause of enteric diseases [4]. Frequent isolation of *Campylobacter* by surveillance programs and primary research surveys in Canada and around the world have highlighted chicken as an important source of *Campylobacter* [5]–[9]. More recently Canadian antimicrobial resistance surveillance has detected regional increases in fluoroquinolone-resistant *Campylobacter* in chicken, which is a public health concern as fluoroquinolones are a class of antimicrobials considered “critically-important” to human medicine [10].

*Campylobacter* risk management in poultry starts at the farm-level to reduce downstream dissemination [11]. Many risk factors for broiler colonisation with *Campylobacter* and the emergence of antimicrobial resistant *Campylobacter* have been identified in the literature: environment, livestock, pests, wildlife, equipment and farm workers, catching crews, antimicrobial use (AMU) and to a lesser extent vertical/pseudovertical transfer [12]–[15]. There has been an increasing amount of research done on *Campylobacter* in broilers and much advancement in the microbiological and molecular methods for identification and quantification of *Campylobacter* in a variety of samples. Previously conducted reviews on *Campylobacter* sources for poultry identify and prioritize control options, inform *Campylobacter* performance objectives and summarize evidence for vertical and horizontal transfer of *Campylobacter* in the poultry industry [5], [12], [16]–[18]. Studies investigating on-farm sources and risk factors in Canadian broiler flocks are limited, thus this systematic review (SR) examines the global evidence for sources and risk factors of *Campylobacter* and antimicrobial resistant-*Campylobacter* in broilers at the farm-level (excluding processing) to determine prevalence estimates and characterise epidemiological linkages described in available primary research.

Systematic review (SR) is a transparent and replicable methodology to identify, critically appraise and synthesize the literature on a clearly formulated question [19], [20]. Meta-analysis (MA) is a statistical method to combine results from similar studies identified in a SR, that measure the same outcome, into an overall average estimate of effect [20]. This SR tabulates the global evidence and presents meta-analytic and molecular summaries on sources of *Campylobacter* for broilers, which has not been done to our knowledge. In addition, the SR updates 7 years of research from a previous SR [12] and identifies agreement or changes in our research knowledge since other reviews and predictive models have been conducted [13], [16], . Results from this SR will be used to identify potential sources and control points/measures that may be relevant to the Canadian broiler industry to reduce *Campylobacter* and antimicrobial resistant *Campylobacter* on-farm. Knowledge gaps and future research priorities to address knowledge gaps for *Campylobacter* in broilers are also highlighted.

The objectives of this present SR-MA are: 1) to identify and characterise the global evidence for sources of *Campylobacter* and/or resistant *Campylobacter* in broilers at the farm-level (excluding processing), 2) to estimate the average prevalence and agreement across epidemiological studies for various sources and, 3) assesses laboratory methods used to isolate and characterise *Campylobacter* in primary research. For the purpose of this SR, a “source” pertains to any potential exposure route (e.g. environment, animals, water, vertical transfer) for *Campylobacter* infection in broilers identified by microbial detection or as a risk factor.

## Methods

### Team, question, protocol and definitions

The systematic review research team included expertise in the following area: microbiology/molecular microbiology, poultry production and processing, food safety, epidemiology, bibliographic, and synthesis research (e.g. systematic review and meta-analysis). The team defined the broad research question to include all French and English primary research investigating any source of on-farm *Campylobacter* spp. for broiler chickens during grow out. General categories included sources related to vertical transmission, domestic and wild animals, humans, water, environment and farm equipment. We were interested in capturing any potential source of *Campylobacter* infection to broilers identified by microbial detection and/or as a risk factor; all study designs and all types of broiler production (e.g. conventional, organic, and free-range) were included. *A priori* developed and pre-tested SR protocol included the study question, sub-questions, definitions, procedure for literature search, study inclusion/exclusion criteria and checklists for conducting relevance screening, basic characterization, methodological assessment and data extraction on relevant primary research, following the general principles of SR methodology [19].

### Search strategy

A broad list of search terms was developed by the research team in order to retrieve all citations pertaining to sources of *Campylobacter* in broiler chickens. The searches were conducted by combining the following terms: *Campylobacter* (n = 1 term) AND broilers (n = 5) AND on-farm sources, e.g., animals, environment (n = 63). At this stage, no restrictions or filters were imposed in terms of study design, publication language, origin of study, and publication date. The search was conducted in four electronic bibliographic databases (PubMED, Scopus, Current Contents, Food Safety and Technology Abstracts) to increase the probability of identifying all potentially relevant publications. The literature search was performed in January 2012. Citations retrieved from all databases were imported into a reference management software (“RefWorks-COS”, ProQuest LLC, Ann Arbor, MI) and de-duplicated. Search verification included hand-searching of reference lists of review articles [12], [16], [22] and 3 recent relevant primary research articles [23]–[25].

### Abstract and article-level relevance screening and study characterization

**Figure 1:**
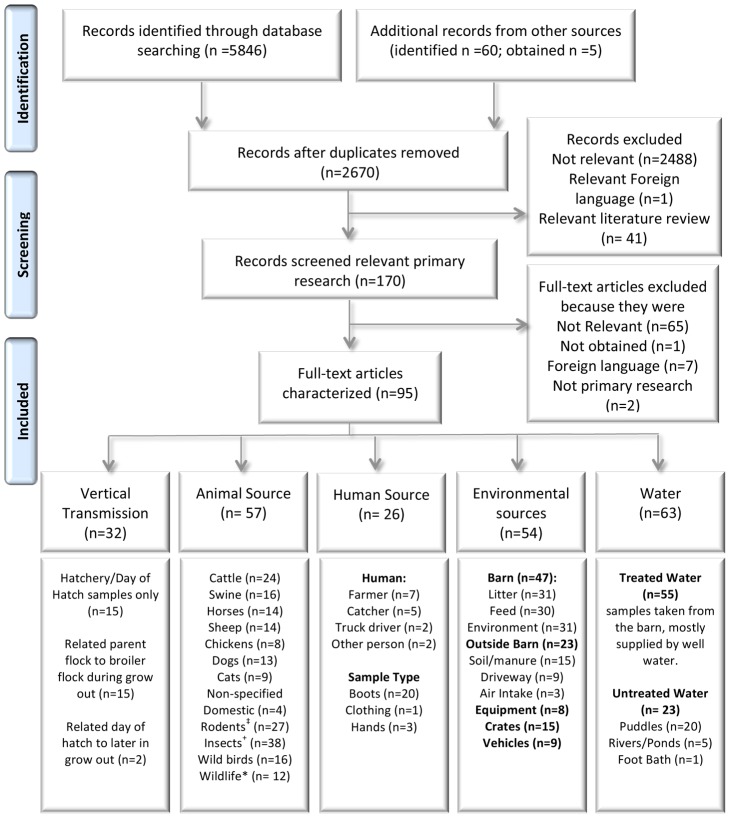
Flow of abstracts and articles through different steps of the systematic review. 95 articles were relevant. ‡rodents identified were mainly mice, also included voles, rats and guinea pig. + Insects identified were mainly flies and beetles with only a few examining mealworm, cockroach, caterpillar and snails. *Wildlife identified included rabbit, raccoon, possum, skunk, deer, mink, fox, hedgehog, shrew and Pukeko.

Through initial abstract-based screening (Figure 1) potentially relevant primary research in English or French investigating *Campylobacter* spp. in broiler chickens on-farm and sources of *Campylobacter* spp. of interest were identified (Appendix S1a). Non-primary research studies (e.g. narrative reviews) or primary research studies outside of the scope were intentionally excluded. All citations deemed relevant at the abstract-based screening level were procured as full articles. At the next level, the full paper was used to confirm relevance, assess and categorize the main themes (e.g. vertical transmission, animal, human, water, environmental, or equipment sources of *Campylobacter* on-farm) and various descriptive characteristics (Appendix S1b). During the second level screening articles were assessed for two exclusion criteria: 1) minimum sufficient reporting of data and, 2) replicable laboratory protocols. Minimum sufficient data was defined as reporting of sufficient (e.g. proportion and number of samples) extractable raw or unadjusted data. For studies which employed multivariable modeling, effect estimates with reported sample size and measure of variability or exact *p*-values were considered essential. Studies were also excluded if the laboratory methods and materials sections lacked sufficient detail or did not reference a paper with sufficient detail to permit replication. Both screening levels were conducted by two independent reviewers using standardized and pre-tested checklists. Reviewer agreement (κ≥0.8) was evaluated using 30 abstracts and 5 full articles for abstract and full paper screening, respectively. Conflicts were resolved through consensus between respective reviewers and if this was not possible, by a third team member.

### Risk of Bias, GRADE and Data Extraction

On prioritized subsets of data the review implemented further assessment of groups of studies (Appendix S1c, S1d). A direct modification of the Risk of Bias and GRADE criteria endorsed by the Cochrane collaboration was used to assess each study and evaluate the GRADE for groups of similar studies [26], [27]. The risk of bias assessment aims to assess the internal validity of the study which informs one of the GRADE criteria. GRADE criteria are summarized across groups of like studies to indicate the level of confidence in the current evidence. Essentially the one to four star grading system indicates that future studies are **** unlikely to change the conclusions of the current evidence; *** may have some impact on the conclusions; ** will likely change the conclusions; * the current evidence is very weak [28]–[30].

The data extraction part of this tool (Appendix S1e) aimed to efficiently capture the pertinent information and results needed for meta-analysis. Any studies where it was noted at the full paper relevance and characterization step that there was “no data to extract” did not progress to this level of evaluation.

### Study management and data analysis

All SR stages; relevance screening at the abstract and paper level, methodological assessment and data extraction were conducted in a web-based electronic systematic review management software DistillerSR (Evidence Partners Inc., Ottawa, Canada). The data were analyzed descriptively (STATA 12, Microsoft Excel 2010) followed by random effect meta-analysis (MA) of selected sub-groups of data in Comprehensive Meta-analysis Software V2 (Biostat, Englewood, NJ). Where appropriate, homogenous meta-analyses with more than 10 lines of data were evaluated for publication bias by Begg's and Egger's test [31], [32]. If publication bias was detected, Duval and Tweedie's trim and fill method was used to estimate the potential impact on the estimate and conclusions of the MA. Results with high heterogeneity are still reported in this manuscript as the authors felt that these results were more informative than reporting a range of results. However, we caution readers to use heterogeneous results carefully as the source of heterogeneity is unknown and future research may dramatically change the results of the MA.

## Results

### Description of studies

The electronic search yielded 2670 citations after de-duplication, including 60 citations identified through search verification (Figure 1). Out of 2530 abstracts excluded through RS1, 2488 were not relevant to sources of *Campylobacter* spp. in broiler chickens, one was considered relevant but in a foreign language and 41 were considered relevant literature reviews or commentaries. Among 170 abstracts considered potentially relevant after abstract screening, five had been identified only through search verification. At the second level screening of the full paper (n = 170), an additional 75 articles were excluded for reasons described in Figure 1.

The 95 articles (Appendix S2) evaluated for descriptive characteristics were published in English and originated from United States of America (USA)/Canada 19.1%, Europe 63.8%, South and Central America 2.0%, Asia 8.5%, Africa 1.0% and Australia and New Zealand 5.3%. The first relevant study was published in 1983 and 75% of the studies captured were published 2000–2012. Studies were mainly observational (92%), with the most common study design being cross-sectional (42.4%) and cohort (29.3%). Thirty seven (38.9%) studies included molecular epidemiology data with respect to the *Campylobacter* spp. samples taken. Conventional broiler production was the focus of 89% of studies, with 4.0% and 6.0% reporting results for organic and free-range production systems, respectively and 3% did not report production type. All studies investigated *Campylobacter* spp. in broiler chickens however, only 3 of these also reported antimicrobial susceptibility results and 9 reported antimicrobial drug usage in the flocks studied.

There were four exclusion criteria in our methodological assessment at the second level of screening with the full paper. Six studies were excluded from further analysis because they did not provide sufficient data for at least one outcome of interest based on the guidelines provided to the reviewers. No studies were excluded based on the other three criteria. Eighty-seven percent appropriately described the methods to allow the study to be reproduced with the remaining 13% considered sufficient because the missing details were referenced to other sources. Seventeen percent sufficiently described the *Campylobacter* spp. isolation and characterization methods and 83% of the 95 studies had referenced pertinent information in their laboratory methods. The final criterion, all 8 experimental studies (e.g., control and quasi-experimental trials) were considered to have selected an appropriate control group.

### Assessment of farm sources of *Campylobacter* spp

Studies were divided into categories based on the source(s) of *Campylobacter* spp. investigated. Many studies investigated more than one: vertical transmission (32 studies), domestic and wild animal sources (57), human sources (26), environmental sources and equipment (54) and water (63) (Figure 1). The outcomes reported are summarized in Tables 1 to 5 and include 3 types of outcomes: 1) *Campylobacter* spp. prevalence information from all source categories, 2) Association measure [Odds Ratio (OR)], reporting the odds that a sample would be *Campylobacter* -positive if it originated from a farm where the broiler flock was *Campylobacter* -positive, 3) Association measure (OR) reporting the odds that a broiler flock is *Campylobacter* -positive when a risk factor (e.g., animal, pest or environmental) is present. Studies that used molecular methods to link broiler isolates from sources were also noted.

**Table 1:** Summary of meta-analysis, genotyping results and GRADE for domestic animals on farm or adjacent to farms.

Category	# studies (lines of data)	MA # studies (lines of data)	Outcome  :	Risk Factor	MA results (95% CI)	Heterogeneity (I^2^)	Molecular linkage to broiler flock (# of studies)§			Adapted GRADE¥
			Prevalence				#tested	Reported matched	Reported not related	
			Odds (sample)							
			Odds (questionnaire)							
Adjacent Broilers	8 (13)	6 (9)	Prevalence		46.2% (26.6–67.0)	High (82.4%)	8	8	NA	**
		3 (5)	Odds (sample)	Adjacent broilers	124.78 (3.6–288.2)	High (79.7%)				**
Laying Hens	3 (3)	2 (2)	Prevalence		87.1% (78.4–92.6)	Low (9.84%)	1	1		**
Cats	2 (2)	1 (1)	Prevalence		7.1% (1.0–37)		1	NA	1	**
Cattle	15 (22)	10 (14)	Prevalence		50.0% (43.1–56.8)	Low (11.1%)	12	8	1	***
		3 (3)	Odds (sample)	Cattle	0.71 (0.30–1.73)	Moderate (48.4%)				**
		2 (2)	Odds (questionnaire)	Cattle on farm	1.44 (0.97–2.15)	Low (0.0%)				**
Dogs	8(10)	6 (8)	Prevalence	Dogs	28.0% (9.4–59.4)	Moderate (66.7%)	7	1	4	**
		2 (2)	Odds (questionnaire)	Dogs on farm	1.76 (1.23–2.56)	Low (0.0%)				*
Horses	7 (9)	3 (4)	Prevalence		29.4% (14.8–49.9)	Low (0.0%)	7	1	5	**
		1 (1)	Odds (questionnaire)	horses on farm	0.90 (0.64–1.27)	Low (0.0%)				*
Pigs	7 (8)	6 (7)	Prevalence		62.4% (37.8–81.9)	High (84%)	5	1	1	**
		1 (1)	Odds (sample)	Pigs	2.03 (0.30–13.9)	NA				*
		3 (3)	Odds (questionnaire)	Pigs on farm	1.44 (0.92–2.27)	Low (0.0%)				**
Sheep	7 (9)	4 (5)	Prevalence		29.3% (5.6–74.5)	High (85.5%)	6	2	2	**
		2 (2)	Odds (questionnaire)	Sheep on farm	1.55 (0.85–1.56)	Moderate (42.9%)				*
Animals, unspecified	5 (6)	5 (6)	Odds (questionnaire)	Animals on farm	2.02 (1.07–3.79)	Moderate (44.0%)	1	1		**
Livestock, unspecified	2 (2)	2 (2)	Odds (questionnaire)	Livestock on farm	0.61 (0.35–1.05)	Low (0.0%)				*
Other birds (e.g., bantam)	1 (1)	1 (1)	Odds (questionnaire)	Other birds on farm	2.10 (0.94–4.68)	Low (0.0%)	1			*


Outcomes include prevalence of *Campylobacter* ssp. in the sample, association measures (Odds Ratio based on sampling and Odds Ratio based on questionnaires to establish the presence or absence of risk factors.).

¥ GRADE, Adapted Grading of Recommendations Assessment, Development and Evaluation (GRADE) criteria, see methods for details.

§ all 37 studies that reported molecular epidemiologic data, number tested include all that obtained samples (positive and negative culture).

MA- meta-analysis, 95% CI – 95% confidence interval, I^2^- measure of heterogeneity in the meta-analysis, NA- Not applicable or not isolated or no matching genotype found or not reported.

**Table 2:** Summary of meta-analysis, genotyping results and GRADE for hygiene barriers, pests, wildlife and humans.

Category	# studies (lines of data)	MA # studies (lines of data)	Outcome  :	Risk Factor	MA results (95% CI)	Heterogeneity (I^2^)	Molecular linkage to broiler flock (# of studies)§			Adapted GRADE¥
			Prevalence				#tested	Reported matched	Reported not related	
			Odds (sample)							
			Odds (questionnaire)							
Hygiene barriers	3 (6)	3 (6)	Odds (questionnaire)	Use of hygiene barriers around broiler barn	0.23 (0.12–0.44)	Low (0.0%)				**
Insects/Beetles	8 (13)	5 (10)	Prevalence		28.9% (12.1–54.8)	High (73%)	6	3	3	**
Insects/Fly	9 (12)	7 (10)	Prevalence		7.1% (1.6–26.0)	High (98%)	3	3	3	*
Insects/all	16 (27)	8 (15)	Prevalence		11.8% (4.3–28.2)	High (88%)	10	4	5	**
Insects/litterbug	1 (3)	1 (1)	Prevalence		83.3% (19.4–99.0)		1	1		**
Rodents	18 (26)	7 (10)	Prevalence		49.6% (24.0–75.5)	High (88.7)	10	2	4	**
		5 (5)	Odds (questionnaire)	Presence of rodents/insects	2.38 (1.34–3.27)	Moderate (51%)				**
Wildlife/deer	1 (2)		NA		Culture negative		1			*
Wildlife/racoon	1 (1)		One isolate		Isolated once		1		1	*
Wildlife/rabbit	1 (1)	1 (1)	Prevalence		10.0%(0.6–67.4)		3		2	*
Wild Birds	9 (15)	4 (9)	Prevalence		37.2%(28.4–47)	High (71%)	9	3	4	**
Humans, Boots	13 (2)	9 (20)	Prevalence		14.1% (8.7–22.0)	High (69%)	10	9		**
		3 (4)	Odds (sample)	Worker boots positive	39.8 (8.0–196)	Low (13.6%)				
Humans, hands	3 (5)	3 (5)	Prevalence		11.6% (6.9–18.9)	Moderate (48%)	2	2		**
Humans, lunch bag	1 (1)	1 (1)	Prevalence		60% (29.7–84.2)		1	1		*


Outcomes include prevalence of *Campylobacter* ssp. in the sample, association measures (Odds Ratio based on sampling and Odds Ratio based on questionnaires to establish the presence or absence of risk factors.).

¥ GRADE, Adapted Grading of Recommendations Assessment, Development and Evaluation (GRADE) criteria, see methods for details.

§ all 37 studies that reported molecular epidemiologic data, number tested include all that obtained samples (positive and negative culture).

MA- meta-analysis, 95% CI – 95% confidence interval, I^2^- measure of heterogeneity in the meta-analysis, NA- Not applicable or not isolated or no matching genotype found or not reported.

**Table 3:** Summary of meta-analysis, genotyping results and GRADE for catching equipment and other environmental-type samples.

Category	# studies (lines of data)	MA # studies (lines of data)	Outcome  :	Risk Factor	MA results (95% CI)	Heterogeneity (I^2^)	Molecular linkage to broiler flock (# of studies)§			Adapted GRADE¥
			Prevalence				#tested§	Reported matched	Reported not related	
			Odds (sample)							
			Odds (questionnaire)							
Catching equipment	3 (7)	3 (7)	Prevalence		35.3% (30.4–40.6)	High (71%)	3	3		*
Crates	12(16)	13 (19)	Prevalence		48.1% (44.7–51.6)	High (89%)	12	11		*
Transport equipment	4 (11)	4 (11)	Prevalence		27.3% (24.8–29.9)	High (87%)	3	3		*
Forklift	3 (3)	3 (3)	Prevalence		21.1% (14.0–30.6)	Low (0%)	2	2		*
Tractor	3 (3)	3 (3)	Prevalence		15.2% (9.4–23.5)	Low (0%)	2	2		*
Feed	22 (32)	13 (23)	Prevalence		4.2% (2.3–7.6)	Low (26%)	11	2	8	***
Litter	4 (8)	3 (3)	Prevalence	Clean	10.4% (2.1–38.8)	Moderate (62%)	9†	4	4	**
	3 (3)	3 (3)	Prevalence	Day 0–1 broilers	8.1% (1.6–31.5)	High (79%)				**
	3 (6)	3 (4)	Prevalence	Day 7–14 broilers	10.5% (3.1–29.6)	High (67.5%)				**
	10 (14)	7 (8)	Prevalence	Day 21–42 broilers	11.1% (7.5–16.2)	High (90%)				**


Outcomes include prevalence of *Campylobacter* ssp. in the sample, association measures (Odds Ratio – samples and Odds Ratio – questionnaire).

¥ GRADE, Adapted Grading of Recommendations Assessment, Development and Evaluation (GRADE) criteria, see methods for details.

§ all 37 studies that reported molecular epidemiologic data, number tested include all that obtained samples (positive and negative culture).

† all age sub-groups.

MA- meta-analysis, 95% CI – 95% confidence interval, I^2^- measure of heterogeneity in the meta-analysis.

**Table 4:** Summary of meta-analysis, genotyping results and GRADE for exterior and interior barn environment.

Category	# studies (lines of data)	MA # studies (lines of data)	Outcome  :	Risk Factor	MA results (95% CI)	Heterogeneity (I^2^)	Molecular linkage to broiler flock (# of studies)§			Adapted GRADE¥
			Prevalence				#tested§	Reported matched	Reported not related	
			Odds (sample)							
			Odds (questionnaire)							
Air Samples	11 (21)	10 (20)	Prevalence		7.3% (3.5–14.6)	High (83%)	7	5	1	**
		1 (1)	Odds (sample)	Air sample	24.05 (1.1–553.3)					*
Concrete around shed and driveway	5 (7)	4 (6)	Prevalence		21.9% (11–38.8)	High (78%)	5	5		*
		1 (1)	Odds (sample)	Concrete	1.80 (0.06–54.6)					*
Shed exterior	7 (13)	6 (11)	Prevalence		18.3% (12.7–25.7)	High (91%)	6	5	1	**
		2 (4)	Odds (sample)	Shed exterior	4.96 (3.49–7.04)	Low (0%)				**
Shed Interior	29 (84)	25 (56)	Prevalence		15.0% (11.5–19.3)	High (93%)	16	13	1	**
		4 (8)	Odds (sample)	Shed interior	9.95 (4.76–20.78)	High (69%)				**
Dead stock	3 (5)	2 (4)	Prevalence		15.1% (2.2–58.2)	Moderate (64%)	3	2		**
		1 (1)	Odds (sample)	Dead stock	3.46 (12.0–100.6)					*
Foot bath	1 (3)	1 (3)	Prevalence		58% (25.0–85.0)	High (74%)	1	1		*
Manure pile	1 (3)	1 (3)	Prevalence		13.7% (4.4–35.1)	Low (9%)	1		1	*
		1 (1)	Odds (sample)	Manure pile	1.67 (0.04–63.8)					*
Soil	3 (9)	2 (6)	Prevalence		14.5% (6.8–28.2)	Low (0%)	3	3		**
		2 (3)	Odds (sample)	Soil	0.57 (0.08–4.03)	Low (0%)				**
Puddle/ditch	12 (25)	9(20)	Prevalence		21.4% (16.6%–27.0%)	Low (26%)	11	7	2	**
		4 (5)	Odds (sample)	Puddle/ditch	1.6 (0.71–3.54)	Low (0%)				**
Pond/river	2 (3)	2 (3)	Prevalence		64.8% (7.3–79.0)	Moderate (50%)	2	0	1	*


Outcomes include prevalence of *Campylobacter* ssp. in the sample, association measures (Odds Ratio based on sampling and Odds Ratio based on questionnaires to establish the presence or absence of risk factors.).

¥ GRADE, Adapted Grading of Recommendations Assessment, Development and Evaluation (GRADE) criteria, see methods for details.

§ all 37 studies that reported molecular epidemiologic data, number tested include all that obtained samples (positive and negative culture).

MA- meta-analysis, 95% CI – 95% confidence interval, I^2^- measure of heterogeneity in the meta-analysis.

**Table 5:** Summary of meta-analysis, genotyping results and GRADE for drinking water and water treatment.

Category	# studies (lines of data)	MA # studies (lines of data)	Outcome  :	Risk Factor	MA results (95% CI)	Heterogeneity (I^2^)	Molecular linkage to broiler flock (# of studies)§			Adapted GRADE¥
			Prevalence				#tested§	Reported matched	Reported not related	
			Odds (sample)							
			Odds (questionnaire)							
Drinking water from the barn	31 (56)	18 (37)	Prevalence		10.8% (7.8–14.7)	High (88.5%)	21	12	7	**
		4 (5)	Odds (sample)	Drinking water from the barn	6.19 (1.06–36.24)	Moderate (50%)				**
Municipal Water		8 (10)	Odds (questionnaire)	Municipal Water vs. well water	1.36 (0.7–2.61)	High (77%)				**
Acidified Water		2 (2)	Odds (questionnaire)	Acidified Water vs. well water	1.84 (0.43–7.91)	Moderate (67%)				*
Filtered Water		1 (1)	Odds (questionnaire)	Filtered Water vs. well water	0.45 (0.16–1.28)	Low (0%)				*
Treated Water		3 (3)	Odds (questionnaire)	Treated Water vs. well water	1.02 (0.51–2.07)	Low (0%)				**
Untreated Water (lakes)		1 (1)	Odds (questionnaire)	Untreated water vs. municipal water	3.42 (1.01–11.55)					


Outcomes include prevalence of *Campylobacter* ssp. in the sample, association measures (Odds Ratio based on sampling and Odds Ratio based on questionnaires to establish the presence or absence of risk factors.).

¥ GRADE, Adapted Grading of Recommendations Assessment, Development and Evaluation (GRADE) criteria, see methods for details.

§ all 37 studies that reported molecular epidemiologic data, number tested include all that obtained samples (positive and negative culture).

MA- meta-analysis, 95% CI – 95% confidence interval, I^2^- measure of heterogeneity in the meta-analysis.

Overall, the prevalence of *Campylobacter* in broilers increased with the age of the flock (data not shown) with peak shedding occurring between days 21 to 42 of grow out.

#### Vertical transmission

This route was investigated in 32 studies included in this SR, and is defined as any study that examined the transmission of *Campylobacter* spp. from broiler breeder flocks to their offspring. The number of studies investigating transmission was the same in Europe and North America and all studies were observational in design: cohort, cross-sectional and prevalence. The overall GRADE of the evidence for these 32 studies was two stars (results not shown), indicating we have little confidence the overall conclusions from current research will remain the same with the addition of future studies. Observational study designs are downgraded compared to highly controlled trials, and in this case additional down-grading occurred due to conflicting results between studies. The 32 studies captured on vertical transmission were all published in peer-reviewed journals, 18 studies were published since (n = 10) or were not captured (n = 8) by Adkin et al [12]. Of these 32 studies, only 11 suggest their results support the possibility of vertical transmission. One of two studies [33], [34] within this subset investigating antimicrobial resistant *Campylobacter* supported the potential for vertical transmission.

From 32 studies, 15 related the breeding flock to subsequent progeny *Campylobacter* status between hatching and the pre-slaughter stage. Fifteen studies only sampled at the hatchery and/or day-old chicks for *Campylobacter*. Twenty seven of 32 studies attempted to isolate *Campylobacter* in birds less than two weeks or in hatch material, however only five studies successfully cultured positive results from hatch materials [35], day old chicks [36]–[38] and one week old chicks [39]. Very few studies followed sufficiently similar designs, or reported comparable results and 3 studies did not have extractable data, thus MA was not possible for these results.

Other evidence investigating vertical transmission included risk factor and genotyping studies. Two articles reported a significant association to the hatchery [40], [41]; the MA results (forest plot not shown) of these two studies (9 trials) was homogenous and reported an increased odds of 4.8 (95% CI 2.9–8.1), *p*<0.001 depending on the hatchery. Overall, large-scale molecular data for vertical transfer was unavailable. Only 4 of the 13 studies that used genotyping detected at least one broiler flock-matching genotype from breeders by flagellin A gene (*fla*A) short variable region (SVR) sequencing [42], *fla*A Polymerase Chain Reaction (PCR)-Restriction Fragment Length Polymorphism (RFLP) [38], Randomly Amplified Polymorphic DNA (RAPD)/Pulsed Field Gel Electrophoresis (PFGE) [34] and/or by Multi-Locus Sequence Typing (MLST) [43]; the other 9 studies found no matching genotypes and concluded that vertical transmission was unlikely.

#### Animals

Pests, domestic and wild animals investigated are listed (Figure 1). Poultry and livestock were commonly found on-farm or in close proximity to the broiler farms. Recovery rates of *Campylobacter* from adjacent broilers, layers, cattle and pigs were relatively high; in dogs, horses and sheep, recovery rates were low to moderate. The results of animal specific meta-analyses can be found in Table 1. More studies reported the detection of broiler flock matching genotypes [e.g., *fla*A-types, RAPD-types, Amplified Fragment Length Polymorphism (AFLP)-types and PFGE *Kpn/SmaI*-types] from adjacent broiler flocks (n = 8) and cattle (n = 8) compared to pigs (n = 1), sheep (n = 2), horses (n = 1) and dogs (n = 1). Cattle appear to be the most frequently identified non-broiler animal with broiler-flock matching isolates compared to other domestic animals found on-farm/adjacent to farm. In some cases, cattle and dogs are colonized with *Campylobacter* species uncommon to chickens such as *C. hyointestinalis* [43]–[45] and *C. upsaliensies* [46], [47], respectively. Random effect MA of association measures (i.e., odds ratio) indicates that only adjacent broilers are more likely to be *Campylobacter*-positive when the broiler flock in question is *Campylobacter*-positive (i.e., likely bi-directional spread), although *Campylobacter* was found in many domestic animals around the farm, and in few studies, the isolate matched the *Campylobacter* from the broiler flock. The only exception was the presence of dogs on the farm was related to an increased odds (OR = 1.76, 95% CI 1.23–2.56) that the flock would be *Campylobacter* positive, but no isolate matched the broiler flock, Table 1. There was insufficient data available to extensively examine directionality of spread within broiler and livestock production units. One study noted intermittent bidirectional spread between cattle and broilers [48], and 2 studies identified positive animals before chicks were placed in the broiler house [49], [50], suggesting that animals can be a source of *Campylobacter*.

Insects including beetles, flies and pests such as rodents were examined in 21 studies and their prevalence results are shown in Table 2. There was a lot of variability between species depending on the degree of contact with the broilers or their feces and the stage of production the samples were taken. Several studies identified matching genotypes to the broiler flock, but samples were only positive when the broiler flocks were present. The MA of four studies indicated increased odds of *Campylobacter* colonization in the presence of pests (OR = 2.38, 95% CI 1.33–4.27).

The role of wildlife was also assessed and detected few isolates from wild waterfowl (e.g., wild geese) [51] and wild bird (e.g., starlings) [44], [46], [51]–[54] populations, which are common on-farm and carriers of multiple pathogens affecting commercial birds. The average prevalence in these species was 37.2% (95% CI 28.4–47.0) although the data was heterogeneous. The wild bird samples collected yielded negative results in a few studies [43], [48] and where detected, isolates were largely unrelated to broiler flocks [44], [46], [52]; only 3 studies detected flock-matching genotypes on the basis of *fla*A sequencing [53], [54] and combined *fla*A sequencing and MLST (i.e., same clonal complex and sequence types) [51]. There were few samples from other wildlife (e.g., deer, raccoons and rabbits) and those from culture-positive rabbits did not match the broiler flock *Campylobacter* genotypes [44], [48], [54].

Hygiene barriers around the broiler barn to keep out other animals, pests and insects were investigated in three studies, the MA [55] indicated this practice was highly protective (OR = 0.23, 95% CI 0.12–0.44). Two studies examined the association between pest prevention interventions and *Campylobacter* colonization of the broiler flock. Insecticide and rodent control measures were not significantly associated with *Campylobacter* status [56], but fly screens may significantly prevent colonization (*p* = 0.002) or delay colonization (*p*<0.0001) [57].

#### Humans

**Figure 2:**
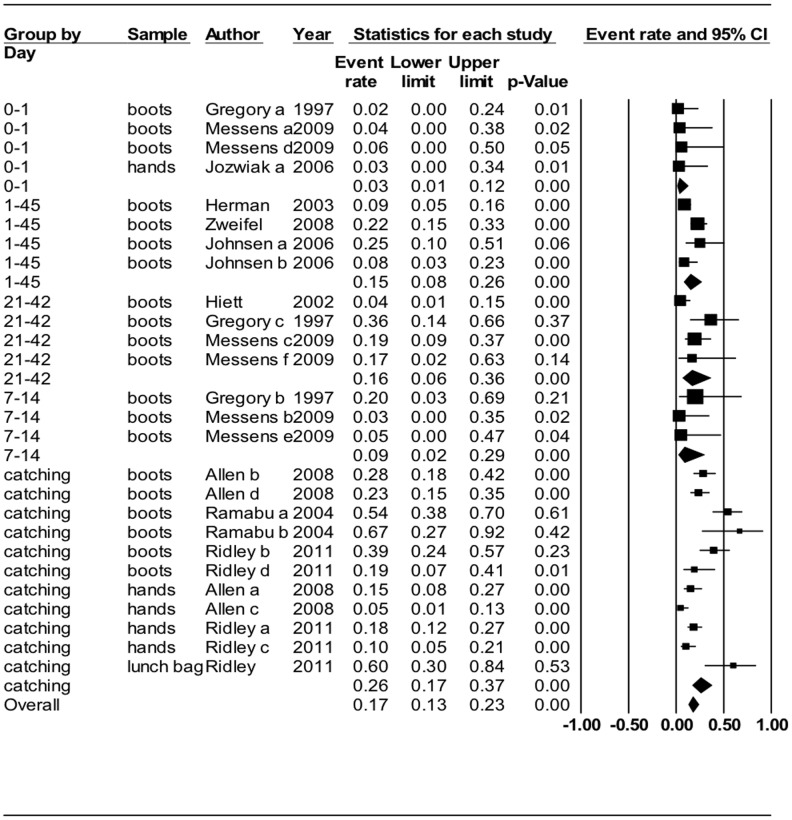
Random effect meta-analysis of *Campylobacter* prevalence by sampling day on human samples. Data from swabs of personal protective equipment, hand surfaces and other personal effects grouped by flock age: day 0–1 (or day of chick placement) I^2^ = 0%, days 1–45 (unspecified age of flock upon sampling) I^2^ = 60%, days 7–14 (early grow) I^2^ = 56%, 21–42 (mid grow to late grow) I^2^ = 0%, and catching (slaughter age) I^2^ = 80%.

The role that people on the broiler farm may play in the contamination of the broiler flock with *Campylobacter* was investigated in 26 observational studies, mainly from Europe, that equally examined the risk of personnel directly involved (e.g., farmer, hired farm workers) and indirectly involved (e.g., catching crew) in flock-rearing. Random effect MA of the prevalence of *Campylobacter* on farm workers' boots, Figure 2, was homogenous and the prevalence increased with the age of the flock: day 0–1, 3.2% (95% CI 0.8–12.0); days 7–14, 8.5% (95% CI 2.1–29.0), and; days 21–42, 16.3% (95% CI 6.4–35.7). In agreement with these data, the prevalence found on the catchers at the end of grow-out was 25.50% (95% CI 16.7–36.9), however this data was heterogeneous. Random effect MA of 3 studies (4 trials) indicates high odds of workers' boots/footwear testing positive for *Campylobacter* if the flock is *Campylobacter* positive (OR = 39.8, 95% CI 8.0–196.0). Nine of 10 studies that genotyped *Campylobacter* isolated from farm workers and catching crew boots/footwear reported that the genotypes matched the broiler flock. Risk factors related to the frequency and number of farm workers were assessed in four studies; conflicting results were found for the number of visits per day [56], [58], and increased odds of the flock being *Campylobacter* positive when there are many farm workers was indicated in two studies [59], [60]. No association was reported between thinning activities and the broiler flock *Campylobacter* status [61].

#### Environmental sources

There were 54 studies that investigated environmental sources, 6 of these were experimental studies mainly focusing on cleaning and disinfection practices. Environmental sources were mainly investigated in Europe (n = 38) compared to other regions of the world (n = 16). Barn, production inputs (e.g., feed and litter), and the equipment to move these inputs were examined in many studies (Figure 1). Sampling of the external environment, the stored manure, vehicles entering the barn area, movable devices (e.g., crates and modules) were also examined in a number of studies.

Heavier equipment, usually kept on-farm such as forklifts and tractors, yielded a lower *Campylobacter* prevalence of 21.1% (95% CI 14–30.6) and 15.2% (95% CI 9.4–23.5), respectively, however there were few studies contributing to these estimates. *Campylobacter* detection from major production inputs such as clean litter and feed was low, Table 3. There was a high prevalence of *Campylobacter* on crates (48.1%, 95% CI 44.7–51.6), on other catching equipment (35.3%, 95% CI 30.4–40.6) and transport equipment (27.3%, 95% CI 24.8–29.9%), all subsets were moderately to highly heterogeneous, Table 3. There is evidence that the genotypes detected from pre-transport crates end up in residual flocks, the remaining birds in the barn, post-thinning [43], [52], [62], [63]. At the abattoir, genotypes from crates were detected from the cloaca [53], [64], [65], feathers and neck skin [64], [66], [67], and in the final carcass/post chill water [53], [67], [68]. None of these studies attempted to trace the main source (e.g., farm or processor origin) of *Campylobacter* contaminating the crates and catching equipment, but the use of the same catching crew was identified as a vehicle for the dissemination of *Campylobacter* within an integrated operation [62]. Other factors such as residual crate contamination and travel time (i.e., related to the time of exposure to the contaminated crate) were investigated in a few studies. In one study, crate washing and immersion in 10% quaternary ammonium compound or 100 ppm hypochlorite solution did not eliminate but slightly reduced the number of *Campylobacter*-positive crates by 40% and 60%, respectively [67]. In another study, exposure to reused crates yielded higher prevalence of *Campylobacter* in the cloaca and neck skin of birds sampled post-transport. Combined cleaning, disinfection and 12 hours of crate/transport equipment drying time was ineffective in reducing the *Campylobacter* load in flocks at the time of slaughter [66]. Travel time to the abattoir and additional data (i.e., pre- and post-transport *Campylobacter* load) were inconsistently reported, hence, it has not been possible to determine the impact of travel time on contamination. Thus, transport equipment and catching personnel pose a risk of transferring new *Campylobacter* to residual flocks and increasing the *Campylobacter* contamination on birds prior to arrival at the abattoir, but these activities were not associated with the *Campylobacter* status of the flock.

There was a wide range of outdoor environmental samples commonly reported; concrete apron and driveways, sample of water from puddles, ditches, and surrounding grass and soil, Table 4. Heterogeneity due to flock age (i.e. association with the *Campylobacter* status of the broiler flock) was investigated in a random effect MA of these outdoor environmental samples (not shown). Flock age did not explain the heterogeneity in the dataset, *Campylobacter* was isolated from day 0 of broiler placement and the prevalence in outdoor samples was relatively constant throughout the grow-out period. However none of the studies were able to demonstrate the direction of spread between flock and environment. Six studies reported detection prior to chick placement/or flock colonization in conventional flocks [43], [48], [65], [69], flocks reared under organic [70] and free-range production systems [71]. Early exposure to the outdoor environment appears to influence the onset of colonization in these production systems [70], [71]. Two studies reported that adjacent flocks remained negative throughout the duration of the growing period despite the presence of *Campylobacter*-positive flocks on the same farm [44], [53], suggesting that biocontainment measures can prevent within-farm dissemination of *Campylobacter*.

Detection of *Campylobacter* in the indoor barn environment (e.g., wall, floor, posts, feeders, drinkers) (15.0%, 95% CI 11.5–19.3) and in air samples (7.3%, 95% CI 3.5–14.6) were moderately and highly heterogeneous, respectively, Table 4. Flock-matching genotypes were detected in indoor environment samples from prior to placement to 24 hours after placement, and more frequently around mid-grow out when flock colonization typically becomes evident.

While carry-over was not an objective of this SR, there were 5 longitudinal prevalence studies and one cohort study that demonstrated carry-over of *Campylobacter* genotypes through two or more flock cycles and also isolated matching genotypes in samples taken in the indoor barn environment demonstrating residual contamination acted as a source for the next broiler cycle [49], [70], [72]–[75].

#### Water

**Figure 3:**
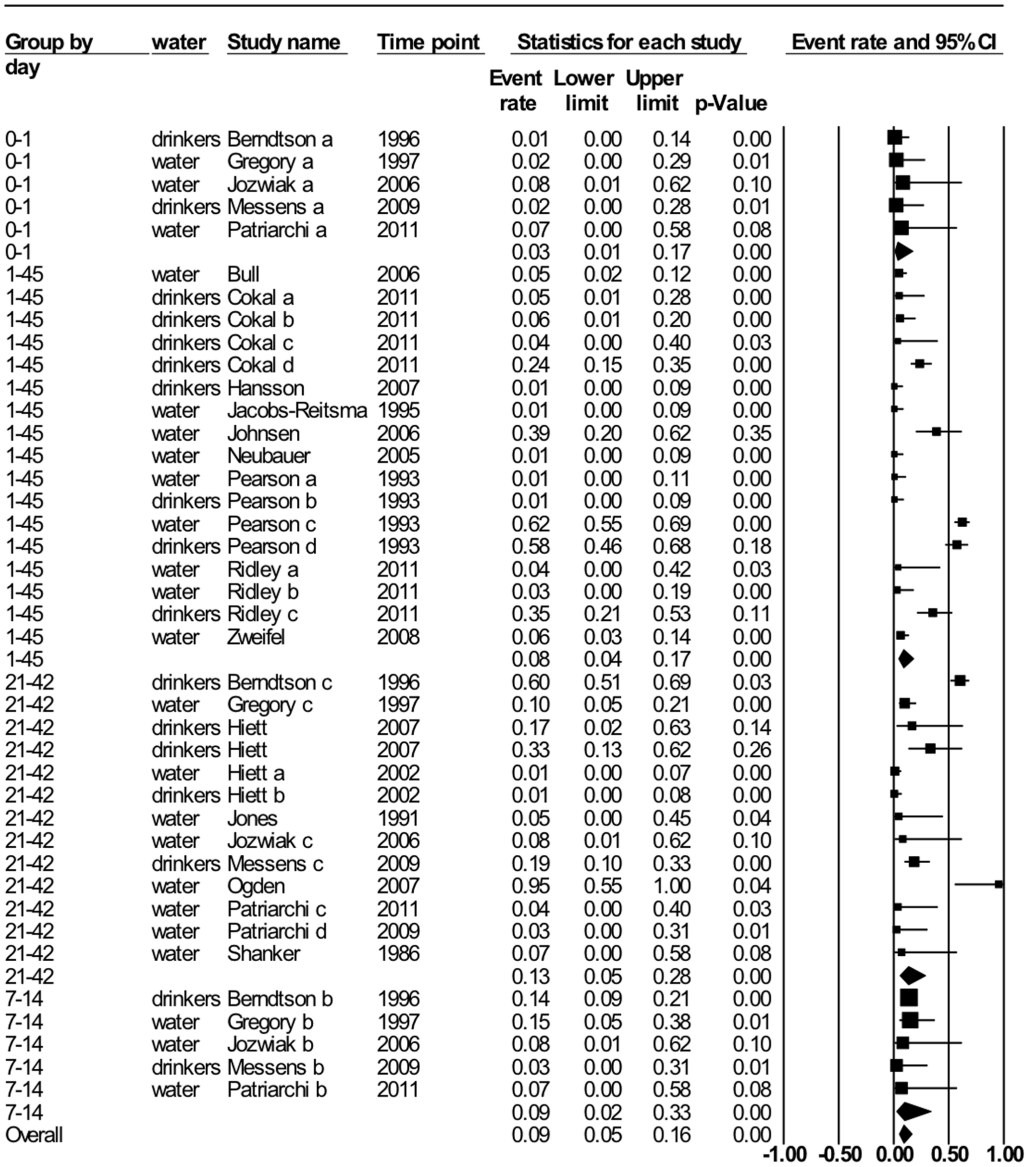
Random effect meta-analysis of *Campylobacter* prevalence by sampling day on water samples. Data from water samples collected from drinkers/water lines or unspecified locations within the barn are grouped by flock age: day 0–1 (or day of chick placement) I^2^ = 0%, days 1–45 (unspecified age of flock upon sampling) I^2^ = 92%, days 7–14 (early grow) I^2^ = 87% and 21–42 (mid grow to late grow) I^2^ = 0%.

Drinking water is a major input into the broiler barn and would be a major source of *Campylobacter* if present. Drinking water samples were taken from a variety of places within the barn and in many cases there is the risk that the *Campylobacter* isolated was from broiler contamination as opposed to the drinking water supply being contaminated. In Figure 3 the temporal MA shows an increase in *Campylobacter* positive water samples with age of the broiler flock. As well, studies that examined the odds of drinking water testing positive in a positive flock was 6.19 (95% CI 1.1–36.2), with moderate heterogeneity (I^2^ = 50%), but the total number of studies was very low. Twelve of 21 studies that genotyped the isolates found flock-matching genotypes in water and water lines in colonized flocks. In two studies, the PFGE patterns of isolates from the drinking line [76] and sequence types from header tank [77] were indistinguishable to isolates from subsequent flocks (i.e., both drinking water system and in bird feces), suggestive of carryover. Less explored niches within the drinking water system such as ciliates and flagellate protozoa also yielded positive *Campylobacter* results [78], [79] and based on the available data drinking water as a source of *Campylobacter* cannot be ruled out. One study also raised the issue of detection limits by culture, as they had immunoflorescent test positives on *Campylobacter* culture negative samples [79]. The studies that examined the water source or treatment of water as a risk factor for flocks testing *Campylobacter* positive did not significantly show an association with the exception of untreated water (e.g. lakes) that had an increased odds of the broiler flock being *Campylobacter* positive OR 3.42 (95% CI 1.01–11.55), Table 5.

### Characterization of antimicrobial-resistant *Campylobacter* and risk factors

There were 10 studies that reported farm-level antimicrobial drug use data. Three studies reported resistance profiles for *Campylobacter* isolates (Table 6), of which two also provided drug-use information. Within these 2 studies, resistant isolates were broiler-flock matched. Idris [34] found resistance patterns in broilers reflective of the medication administered to breeders (i.e., ciprofloxacin, a flouroquilonone antimicrobial). The broiler PFGE and RAPD types matched the breeder genotypes isolated, which suggests vertical transfer occurred. Subsequent exposure of the breeders' descendants to the same antimicrobial resulted in a high Minimum Inhibitory Concentration which implies reduced susceptibility to the antimicrobial. Messens [44] detected isolates from the drinking water, farmer's footwear and other barns, which were resistant to one or more of the drugs that were administered to the flock, suggestive of within-flock (via drinking water) and within farm (via farmers footwear) dissemination of antimicrobial-resistant *Campylobacter*. Skov [80] found nalidixic-acid resistant *C. jejuni* in beetles, but prevalence in beetles and drug use history for the broiler flock were unavailable; the insects were found only while birds were present in the barn and not during downtime.

**Table 6:** Sources of antimicrobial-resistant *Campylobacter*.

Authors	Source/transfer mechanism	Susceptibility test and profiles	Prevalence	Genotyping/Antimicrobial use history/comments
Skov et al, 2004 [80]	Pests (beetle)	Unknown: *Nal*	Not reported	Resistance profile of beetles matched broiler genotypes;Drug use data unavailable;Detected in beetles during growing period but not during downtime.
Idris et al, 2006 [34]	Broiler breeders/vertical transfer	Agar Dilution: *Cip, Cip/Tet*	Descendants of broiler breeders  :*C. jejuni* = 60% *(Cip)* *C. coli* = 23.5% *(Cip)*	Genotypes and resistance profiles of descendants matched the broiler breeder genotypes;Resistance related to drug use in breeders;Detected in descendants of broiler breeders 6 weeks post-medication.
Messens et al, 2009/Herman et al 2003 [44], [45]	Adjacent flock, drinking water, footwear	E-test: *Ery/Tet Tet,Cip/Nal/Tet, Nal/Tet, Cip/Nal*	54% (resistant to 1 or more antimicrobials)	Some genotypes and resistance profiles of adjacent flocks, drinking water and footwear matched broiler flock genotypes;Resistance related to drug use: 12 out of 18 flocks medicated 1–4 times with: Quinolone-Fluoroquinolone (n = 5 flocks); Ampicillin (n = 1); Sulfonamide (n = 1); Fluoroquinolone-Trimethoprim-Sulfonamide (n = 1); Fluoroquinolone-Sulfonamide-Tetracycline (n = 1); Macrolides-Lincosamides-Polypeptide (n = 1); Lincosamides-Aminoglycosides-Quinolone (n = 1); Lincosamides-Aminoglycosides (n = 1);Isolates exhibited resistance to more than one antimicrobial; profile of some isolates detected from the same source changed through time.


Same broiler integrator that supplied the breeder flock in the study.

Ery – erythromycin, Tet – Tetracycline, Cip – ciprofloxacin, Nal – nalidixic acid.

### Assessment of laboratory methods

The laboratory methods captured in the 95 studies were summarized in Table 7. The goal of this exercise was to identify primary isolation protocols, typing/speciation and genotyping tools to link broiler *Campylobacter* from potential sources. This SR found extensive variations in the method of detection for *Campylobacter* and genotyping to establish epidemiological linkages. PCR was the most commonly used subtyping tool, but biotyping and/or serotyping were also used. Agar dilution and E-test methods were used for characterizing resistant profiles. Genotyping was done in 37 studies (38.9%) using one test method (18 studies), 2 test methods (16 studies), and 3 test methods (3 studies). The use of more than one test method enhanced the over-all discriminatory power (i.e., ability to differentiate between strains) or confirmed the genotype groups/clusters, however, concordance (i.e., agreement between results of two independent typing systems [81]) were not well described. Overall, PFGE alone or in combination with other tests (Table 7) was the most frequently used technique, but there were variations in the number of enzymes used (i.e., *Sma*I, 10 studies and *Sma*I+*Kpn*I, 8 studies), and analytical method used to interpret results (i.e., visual observation vs. mathematical). The most common analytical method to determine similarity between genotypes was Dice coefficient (i.e., algorithm that assigns strains to the same group [82]); arbitrary cut-off values ranged from 94 to 99% similarity (i.e., 100% minus 1 to 6%). Genotypes were also assessed if these clustered to the same group, using Unweighted Paired Group Matrix Analysis (UPGMA), Neighbor Joining algorithm and HKY85 method. Most studies interpreted the genotyping results based on the convention for PFGE band interpretation developed by Tenover and others [83] that categorized the genotypes as “indistinguishable”, “closely related”, “possibly related” and “unrelated”. In this study, the term “flock-matching/flock-matched”, “identical” and “indistinguishable” was used synonymously to describe linkages from broilers and the different sources.

**Table 7:** Summary of *Campylobacter* isolation and genotyping protocols.

Recovery steps (%, 95 studies)	Types	# of studies
Farm-collected broiler samples (73.6%)	droppings: fecal, caecal, fecal/caecal swabs	53
	cloacal swabs	13
	boot socks/overshoe	3
	sand sample	1
	other farm-collected but non-broiler samples	(8)
Abattoir level and/or laboratory collected broiler samples (45.2%) 	caecal content/caecal contents swab	30
	cloacal swabs	11
	intestinal pool	2
	Combined with above samples: feathers, neck skin, bile	(4)
Used transport media (25.2%)	Cary Blair	7
	Phosphate Buffered Saline	5
	Maximum Recovery Diluent	4
	Amies Charcoal	4
	Buffered Peptone Water	3
	Used enrichment media to transport specimen	(10)
Incorporated enrichment step (75.7%)	Preston	29
	Bolton	10
	*Campylobacter* Selective Enrichment	10
	Exeter	8
	Hunt's	5
	Less commonly used (1–3 studies): Modified/Double Strength Enrichment, Tryptic Soy Broth, Supplemented Nutrient Broth (5% horse blood), *Brucella*, THAL Campy	10
Culture plating, raw and enriched samples (95.7%)	mCCDA, charcoal- based	30
	CCDA, charcoal- based	12
	Campy-Cefex Agar, charcoal- based	10
	Preston, charcoal- based	10
	*Campylobacter* Selective Agar, blood-free	7
	*Campylobacter* Selective Agar, blood-based	6
	Less commonly used (1–4 studies): Virion, Karmali, Butzler, CAT agar, *Brucella*, Mueller Hinton with Preston Agar, Campy-FDA	16
Direct molecular detection, raw samples or enriched samples (4.2%)	PCR	4
Antimicrobial sensitivity testing (3.1%)	Agar dilution	1
	E-test	1
	Unknown	1
Speciation (81.0%)	PCR	41
	Biotyping and serotyping	24
	PCR combined with biotyping and serotyping	12
Genotyping (molecular linkage) (38.9%)¥		
1 method	*fla*A (sequencing or PCR-RFLP)	5
	RAPD	5
	PFGE	4
	Less frequently used: AFLP, MLST, ribotyping, sequencing of other genes	4
2 methods	*fla*A+PFGE	8
	*fla*A+MLST	4
	Less frequently used: *fla*A+ribotyping, PFGE+RAPD, *fla*A/*fla*B+23S rRNA spacer)	3
3 methods	*fla*A+PFGE+AFLP	2
	*fla*A+PFGE+MLST	1


some studies may have also collected samples on-farm (21 studies collected at the farm and abattoir).

¥ Genotyping to link broiler, animal and environmental type sample matrices; methods were also used for nomenclature/arbitrary genotype assignment.

( ) not counted, mCCDA-modified charcoal cefoperazone deoxycholate agar, PCR-Polymerase Chain Reaction, *fla*A-flagellin A, RAPD- Randomly Amplified Polymorphic DNA, PFGE-Pulsed Field Gel Electrophoresis, AFLP-Amplified Fragment Length Polymorphism. MLST – Multi-locus Sequence Typing, REA-Restriction Endonuclease Analysis.

## Discussion

There has been an increasing interest over the last decade in controlling *Campylobacter* in broiler chickens and minimizing drug-resistant *Campylobacter* in the human food chain. In Canada, campylobacteriosis is the third most common enteric pathogen and recent surveillance data has identified regional increases in Ciprofloxacin-resistant *Campylobacter*, which is a public health concern [4], [10]. *Campylobacter* contaminated broiler meat is a food safety risk and evaluation of the global literature on *Campylobacter* sources is relevant to many countries as general industry-operational factors (e.g., catching, thinning, transport, processing) that may contribute to contamination are similar across countries; though in an integrated system, there may be less complexity in the coordination of monitoring and implementation of control programs throughout the poultry production chain. The Canadian broiler industry is a vertically-coordinated industry (i.e., sectors are closely-linked operationally but not owned by a single company) unlike in many European countries [84] and in the USA [85] which are heavily integrated. However, the product process flow, from hatching egg production to retail in Canada [86], is largely similar to many countries [84], [85]. Canadian broiler hatching eggs/chicks are sourced domestically and approximately 21% are imported from the USA in compliance with trade agreements [87]. This industry practice of international exchange of live products is also practiced in some European countries [88], [89] and may have relevance to the introduction of new *Campylobacter* strains. Broilers are grown out in cycles so there is an all in-all out approach and farms within Canada vary in size and number of barns [90], as in other countries [84], [85].

Similar to other synthesis and evaluation of the literature[12], [13], [16], this SR highlights the need for improved disinfection of the broiler house between flocks and improved biosecurity practices to stop the potentially self-perpetuating cycle of flock contamination. The meta-analysis of quantitative outcomes in this SR improves our understanding of the degree of variation across studies and covariates that account for some of the heterogeneity. The increasing use of molecular methods over the last decade has been highlighted in this review and adds more evidence to substantiate the overall findings and recommendations. This information, used in conjunction with context specific knowledge of the local broiler industry, can identify critical control points for prevention of broiler flock *Campylobacter* contamination.

One of the findings from this SR is that *Campylobacter* can be found on the farm prior to a new flock arriving at the broiler house. Inadequate cleaning and disinfection and short downtime of the broiler house between flocks may be a major source of *Campylobacter* carryover as studies confirmed *Campylobacter* in dust swabs from the interior of the barn and the drinking water system prior to or during flock placement [43], [44], [70], [71], [91]. The cycle of contaminated flocks is self-perpetuating if chicks are being placed in an already contaminated environment, in this SR 83% of the studies that reported carryover concluded persistent *Campylobacter* genotypes could be identified through several cycles [49], [70], [72]–[75]. In agreement with Adkin et al [12] and Newell et al [16] inputs such as clean litter and feed into the barn were not likely sources of *Campylobacter* to young broilers. Drinking water was found to be a potential vehicle for flock-level contamination; in agreement with our meta-analyses the consumption of untreated water was associated with an increased odds of a *Campylobacter* positive broiler flock. There was some evidence for vertical transmission or pseudovertical transfer (e.g. egg surface contamination) however it seems that this research is still hampered by the sensitivity of isolation and genotyping techniques, which will be discussed later. Meta-analysis of two studies associated the hatchery source to *Campylobacter* colonization at the broiler level (OR = 4.8) [40], [41] and Idris et al demonstrated identical resistance patterns in the breeders and broilers [34]. Previous reviews that looked at vertical transmission concluded this was a low risk exposure route for *Campylobacter* in broilers [12], [13], however based on the evidence identified in this SR there is evidence that vertical transfer occurs and due to well documented isolation issues, we cannot conclude on the relative importance of this source compared to others. There have been many investigations into potential biological mechanisms related to the survival of *Campylobacter* from egg to post-hatch stages [18], but more research is required to investigate this potentially under-recognized transmission route. It appears that any of these sources could result in an infected broiler flock as only a few susceptible birds are sufficient to result in flock-level colonization by the end of grow out [21], [92].

Domestic animals and their environment were positive for *Campylobacter* in several studies, but they had very small sample size and often these isolates did not match the *Campylobacter* that subsequently colonized the flock. This SR-MA identified adjacent broiler flocks (n = 8 studies) as a source of flock-matching genotypes (8/8 studies) and the MA (OR = 124.78) suggests that the presence of adjacent broilers may pose a significant risk for *Campylobacter* transmission to a new flock in agreement with other synthesis papers [16], [21]. The same was not seen for other animals (e.g., cattle) on the farm, despite high prevalence and the detection of flock-matching genotypes in 66% (8/12) of studies. Overall, there was very little wildlife evidence and the 9 wild bird studies reported an average prevalence of 37.2% with flock matching genotypes reported in 33% of studies and no epidemiological analysis. These were also considered low risks for *Campylobacter* infection in broilers in the UK [12]; and were not prioritized for intervention relative to other vectors and production factors in recent guidance documents [13]. There is Canadian surveillance evidence that indicates where farming is dense and species are diverse, *Campylobacter* in particular, can be very high [93] and is likely being shared among operations [94], [95]. The potential for transmission of *Campylobacter* via other infected animals or pests highlights the importance of biosecurity, including hygiene barriers between animal production units. A number of biosecurity measures have been highlighted in previous reviews [12], [13], [16] including restrictions of other animals on the farm [21] and the use of pest control methods such as fly screens or insecticides [57], however the effectiveness of these interventions still needs to be evaluated for Canadian broiler farms.

Our examination of the evidence on resistance profiles of *Campylobacter* isolated from multiple sources netted three studies; two related the resistance to broiler antimicrobial drug use [34], [44] and one related the antimicrobial-resistant *Campylobacter* to drug use in the breeders [34], implying vertical transfer is possible. With only three studies, it is not possible to draw any conclusions from this data, however understanding the potential transmission routes through the broiler production hierarchy is important to assess the potential role that the international and interprovincial exchange of hatching eggs and chicks may have in the emergence of antimicrobial-resistant *Campylobacter* in Canadian broilers. Farm sources of drug-resistant *Campylobacter* should also be explored such as transmission from beef, dairy and swine herds, although the molecular epidemiology and broiler colonization (e.g., of bovine origin) studies have yet to be conducted.

There were several potential sources of *Campylobacter* that showed a temporal correlation with the flock becoming *Campylobacter* positive. These included pests (e.g. fly and rodents), humans (e.g., visitors, number of workers that enter the barn, frequency of access to barns), farm equipment, and water, which may indicate that these potential sources are actually becoming contaminated by the broiler flock as it is colonized and not vice versa.

Catching crews and independent operators (e.g., truckers, drivers) were identified as a potential source of new genotypes in the flock during late grow-out when *Campylobacter* shedding in the contaminated flocks peaks, increasing the chances of within-farm (i.e., through contaminating the environment) and industry-wide *Campylobacter* dissemination (e.g., through catching and transport equipment). Similarly, flock thinning, practiced by approximately 11% of Canadian broiler producers [96], has been noted as particularly high risk exposure for the remaining flock as *Campylobacter* introduction as low as 2 colony forming units could lead to pen-level colonization within a 7-day period [97]. Katsma et al suggested that banning the practice of thinning may reduce slaughter level *Campylobacter* prevalence [21]. Contamination of the birds on route to slaughter may result in increased external contamination and internal colonization if the birds remain in the contaminated crates for more than 6 hours [43], [64], [66]; bird contamination/colonization may lead to processing plant contamination, which could subsequently contaminate batches of *Campylobacter*-negative flocks [45], thus undermining overall industry-level reduction efforts. Some studies captured in this SR linked crates and catching crew related sources of *Campylobacter* with external contamination of the birds upon entering the abattoir, but none of the studies were appropriately designed to establish the direction of spread. Improvement in biosecurity compliance, such as provision of farm-specific clean boots and clothes to catching crew, was practiced by only 38% of broiler producers in Canada [96]. And improvement in biosecurity interventions such as effective cleaning and disinfection of crates and equipment are needed as current research indicates these methods are ineffective [13] and drying time (<24 hours) may be too short [66]. Factors such as production expectations (i.e., to achieve projected kilograms/quota), related management practices (i.e., thinning) and producer education may have an impact on *Campylobacter* colonization prevention. These are likely to vary widely by region and country, thus the local context should be considered when developing recommendations.

Biosecurity improvements were highlighted throughout this SR and are consistent with the findings of previous reviews [12], [13], [16]. The focus of this review was on sources as opposed to evaluating the effectiveness of various biosecurity interventions, however other reviews have indicated more work needs to be done to assess how effective biosecurity interventions may be in reducing *Campylobacter* positive flocks and within flock prevalence [21]. Current industry and national requirements for biosecurity and the level of compliance by individual producers should be taken into account when modernizing local biosecurity practices. Biosecurity programs generally involve access management (e.g., restriction to rearing areas, hygiene barriers), animal health management and general farm operational/structural improvements to reduce disease transmission [98]. In Canada, biosecurity-associated risks such as rodent control and proximity of the barn to manure piles have been identified as risk factors for *Campylobacter* colonization in turkeys, the same is likely true for broilers [99]. Canadian avian biosecurity recommendations address a number of biosecurity risk reduction practices including installing hygiene barriers, manure management, designating manure storage areas that are distant to the main rearing area, and maintenance and modernization of facilities such as fly screen installation and drainage improvement to reduce pests [98], [100]. Restriction of personnel movement (i.e., in multi-barn facilities) may not be practical when there is a limited number of farm workers hired by the producer, but any of the following management practices could reduce the risk of human movement contributing to *Campylobacter* contamination of the broiler flock. Minimizing the frequency of access by farm workers or following a strict movement protocol (e.g., from young to old flocks in multi-barn facilities), limiting visitors, provision of farm/barn-designated clothing and footwear, decontamination (e.g., proper hand washing), and other structural changes or modernization of facilities (e.g., anteroom and hygiene barriers/fences, separate entrances in multiple barns). In Canada, biosecurity protocols and producer education programs need to be improved as a recently conducted Canadian survey of producers about their knowledge and attitudes toward food safety and good production practices indicates low to moderate compliance for hand washing (36%) and visitors changing clothing before entering barn (50%) [96]. Another Canadian study found farm workers often commit mistakes related to bio-containment (i.e., barn entry and exit procedures) [101]. It is recommended that on-going producer education emphasize the importance of biosecurity, improved compliance with on-farm food safety program recommendations and monitoring within the context of their local broiler industry [98], [100], [102], [103].

Few studies were appropriately designed to establish the direction of *Campylobacter* exchange or to understand the reservoir vs. vector potential of identified sources. In many cases the potential sources are not mutually exclusive e.g., environment, human, equipment could all contribute to both the dissemination and maintenance of *Campylobacter* and once the farm is contaminated, the cycle may be self-perpetuating even with all-in-all-out practices and stringent biosecurity. Forty percent of the studies in this review were cross-sectional, which cannot inform the direction of spread. Additionally, sampling inconsistencies including frequency of sampling, age at sample collection and variations of genotyping techniques (i.e., insensitive to genetic recombination) limited the ability to establish direction of spread in other study designs. Host-preference [48], invasive ability/gut colonization, and competitive performance within the gut [104] are also factors that complicate our understanding of host preference among *Campylobacter*, but are beyond the scope of this SR. The detection of flock-matching genotypes in livestock may be suggestive of an increased proportion of highly host-adaptive subpopulations [105] that result from frequent genetic interchange (i.e., interspecies and intraspecies) [106]. On the other hand, niche segregation among *Campylobacter* has also been described [105] and may explain the presence of highly host-specific/non-flock matching *Campylobacter* subpopulations such as those that colonize wild birds [107]. Host-related genomic markers for source-tracking of *Campylobacter*, has yet to be identified [106]. Future host-association and *Campylobacter* dynamics studies should adopt a more structured sampling methodology and utilize high-performance genotyping techniques that are sensitive to intra-and inter-genomic recombination [108] in addition to MLST [106], [109], [110].

The inability to culture *Campylobacter* from birds less than 2 weeks old presents a major barrier in researching *Campylobacter* in broilers and has led to inconsistent study results within this SR. Several hypotheses have been suggested to explain researchers' inability to isolate *Campylobacter* during the first two weeks of placement. First, *Campylobacter* may be in a non-culturable form as there were several studies that successfully detected *Campylobacter* DNA, but have failed to culture [34], [111]–[113]. Thus, there is a need to explore the use of a more reliable molecular technique for detecting viable or “potentially infectious units” of *Campylobacter* [114] from hatchery and chick samples. Second, different isolation techniques have highly variable sensitivity that may affect results if *Campylobacter* concentration is below the detection limits [113]. Because of the inherently low number of cells in eggs/eggshells, embryos, yolk sac, and neonatal intestines enhanced recovery techniques (e.g., combining membrane filtration and enrichment) [115], [116] need to be explored to improve our detection limits in these samples. Third, the type of sample may be important, for example, *Campylobacter* may not be present in the cecal or fecal samples during early rearing because it is still colonizing the small intestine [34], [117].

The studies captured in this SR used various techniques to sample isolates and evaluate *Campylobacter*. Different sample matrices (e.g., fecal, cecal, cloacal swabs, boot socks), isolation protocols, and genotyping methods were identified and tabulated; all have different sensitivity and specificity, which could influence the prevalence results, genotype diversity and be a source of between study heterogeneity [118]–[120]. Each of the genotyping techniques reported has certain limitations [24], [121], and may not be suitable for longitudinal investigations (i.e., multiple broiler cycles) across wide geographical areas. Two of the older methods RAPD and ribotyping are rarely used now because of typeability issues [122], [123]. Only a few studies identified by this SR used more recent techniques such as MLST. PFGE was the most common among captured studies and is considered the “gold standard” for many priority enteric pathogens. For *Campylobacter*, however, PFGE fingerprints tend to evolve rapidly [83], and interpretation may change based on the number of enzymes used [70], [71]. A Canadian *Campylobacter* outbreak demonstrated that PFGE failed to detect clusters of human cases because the PFGE fingerprints changed during the course of the outbreak [124], same in a Canadian *Salmonella* outbreak [125], which suggests this technique may not be suitable for longitudinal studies (i.e., several broiler production cycles). The use of PFGE, in combination with other methods was described in most studies in this SR. *Fla* typing was the second most frequently used, but it has the potential for recombinational exchange of alleles: unrelated strains may end up with the same *fla* type whereas related strains may end up with different *fla* types [43]. Detailed assessment of the potential impact of these laboratory factors is beyond the scope of this SR, but we have highlighted the variability and quantity of research that is underpinned by each method. Future research should work towards developing an internationally accepted genotyping technique for *Campylobacter* that is inexpensive and meets most of the performance criteria including stability, typeability, reproducibility, discriminatory power and concordance in order to validate the genetic and epidemiological linkages [81].

In conclusion, identifying farm-level sources for contamination of *Campylobacter* in broiler chickens can help identify and prioritize control points and recommendations for biosecurity improvements on-farm and industry-wide and highlights information gaps in understanding the epidemiology of *Campylobacter* in broiler production. This systematic review meta-analysis assessed multiple outcomes (prevalence and risk factors) to identify and summarize the evidence for sources of *Campylobacter* on-farm. The highest risk for contaminating a new flock appears to be a contaminated barn environment due to inadequate disinfection and cleaning, insufficient downtime and the presence of an adjacent broiler flock. Gaps still exist in understanding the role of other domestic and wild animals and vertical transfer in the epidemiology of *Campylobacter* and antimicrobial-resistance profiles of isolates from the different source categories. This study highlights the potential impact of enhanced biosecurity and the need for improved compliance particularly in bio-containment, personnel decontamination measures, and equipment cleaning and disinfection. Further research, such as advanced genotyping methods, sensitive to intra-and intergenomic recombinations are required to improve on-farm source attribution and to provide recommendations for further refinement of food safety programs in Canada.

## Supporting Information

Checklist S1 **PRISMA checklist.**
(DOC)

Appendix S1 **Study Protocol.** Outlines the protocol for relevance and abstract screenings, data characterization, Risk of Bias (RoB), and GRADE, and; data extraction forms.
(DOCX)

Appendix S2 **List of the 95 references used in the study.**
(DOCX)
