# Supplementary material for: A Systematic Review Characterizing On-Farm Sources of Campylobacter spp. for Broiler Chickens
Source: PLoS One. 2014 Aug 29;9(8):e104905. doi: 10.1371/journal.pone.0104905 (PMC4149356; doi:10.1371/journal.pone.0104905)
Supplement: Appendix S1 — Study Protocol. Outlines the protocol for relevance and abstract screenings, data characterization, Risk of Bias (RoB), and GRADE, and; data extraction forms. (DOCX) [file pone.0104905.s002.docx]

**Appendix S1. Study Protocol**

***Appendix S1a. Relevance Tool - Screening Abstracts***

1. Is this primary research in English or French? Y

N primary foreign language

N literature review

N commentary or opinion letter

N Other e.g. economic model

1. Does this research investigate ***Campylobacter spp****.* in **broiler** chickens during the growing period **on-farm**? Y

N

1. If yes to Q1&2: What sources of *Campylobacter* does the research investigate?

i. Vertical transmission from parent flock to progeny Y

ii. Domestic, Wild Animal or Pest sources Y

iii. Human (catchers, visitors) Y

iv. Other Source of *Campylobacter* spp. Y specify____

v. This study evaluates isolation methods for Campy? Y

1. None of above N

(This study is looking at biosecurity that does not relate a vector to flock exposure or contamination or the study is NOT examining sources of *Campylobacter* for broiler flocks.)

**Footnotes:**

All underlined terms are defined in the definitions section of the protocol.

If **No** is answered for Q1 or Q2, please submit form without answering Q3.

------------------------------------------------------------------------------------------------------------

Reviewer Decision

If the reviewer answers yes to questions 1, 2 and 3. The article will be included for further screening and appraisal.

-------------------------------------------------------------------------------------------------------------------------------

***Appendix S1b. Data Characterisation Form***

**Relevance Screening Verification.**

1. Classify the type of article appropriately:
   - Primary research in a foreign language *(*Answer Q1-3 and submit form*. exclude)*
2. Does this research investigate sources of *Campylobacter* spp*.* in broiler chickens during the growing period on- farm? (Relevance verification)
   - Yes
   - No (If NO, Check and submit the form.)
3. Identify the sources of *Campylobacter* spp. investigated (and from where/what the samples were taken from) for this study.

**□ Investigation of vertical transmission of *Campylobacter* from parent flocks**.

Sampled at:

- Parent flock/bird specify: ______
- Hatchery egg samples specify: ______
- Day of hatch chick samples specify: ______
- Broiler flock/bird specify: ______

**□ Investigation of animal/human sources.**

- **Dairy cattle** specify production class, e.g., heifer calf, (veal calf), replacement heifer, lactating cow, dry cow : ______
- **Beef cattle** specify production class, e.g., cow-calf production may include cow, calf, heifer, bull; feedlot production may include light calf, calf, heifer, steer, grower, finisher, market weight;
- **Swine** specify production class, e.g., Swine: sow, barrow, guilt, piglet or nursery pigs, weaners, grower, finisher: ______
- **Other domestic** farmed animals specify (e.g. sheep, goats, etc.): ______
- **Other domestic** animals (e.g. dog, cat) specify: ______
- **Rodents** specify: ______
- **Insects** specify: ______
- **Birds** specify: ______
- **Other wildlife** specify (e.g. deer, squirrels, fox, raccoon): ______
- **Human** source (e.g. Farm worker/visitors/catchers, includes anything worn by the person.) specify: _____

**□ Investigation of Environmental contamination/samples.**

- **Drinking water,** specify: ______
- **Untreated water** on farm, specify: ______
- Samples in the **barn** (anteroom, shed entrance if attached), specify: ______
- Samples on the **farm outside** of the barn, specify: ______
- **Equipment** samples (e.g. shovel, forklift, tractor etc.), specify: ______
- **Transport cages** and crates, specify: ______
- **Vehicle** (e.g. 18 wheeler, farm or visiting trucks etc.), specify: ______

**□ None of above**

- This is ONLY a diagnostic test evaluation
- This study is relevant, but doesn’t fit into above categories, specify:_____
- This study is not relevant (check and submit form.).

**General Study Information:**

1. What is the study design?
   - Longitudinal Prevalence study
   - Prevalence study (survey)
   - Cross-sectional study
   - Controlled Trial (randomised and non-randomised)
   - Molecular Epidemiology study (specify data available) ____________
2. Where did the study occur?

- **North America:** Canada, USA specify: _________
- **South America/Caribbean:** Mexico, Caribbean, and all of South America. specify: _________
- **Europe:** includes, Belarus, Latvia, Ukraine, Estonia, Cyprus and West (including. Iceland and Greenland) specify: _________
- **Asia:** Russia, Turkey, middle eastern countries and east specify: _________
- **Australia:** includes New Zealand and islands Papua New Guinea & south. specify: _________
- **Other:** Africa and Antarctica specify: _________

**Broiler Operation Characteristics:**

1. What type of broiler operation was studied?

- Conventional : _________
- Organic : _________
- Other, specify: _________
- Not stated

1. What type of broiler industry was investigated?

- Vertically integrated operation
- Non-integrated operation
- Other: _________
- Not stated

1. What country did the parent flock originate from? (continent/ country, must be stated in the paper)

- Not stated
- **North America:** Canada, USA specify: _________
- **South America/ Caribbean:** Mexico, Caribbean, and all of south America. specify: _________
- **Europe:** includes, Belarus, Latvia, Ukraine, Estonia, Cyprus & West (including Iceland and Greenland) specify: _________
- **Asia:** Russia, Turkey, Middle Eastern countries and east specify: _________
- **Australia:** includes New Zealand and islands Papua New Guinea and South. specify: _________
- **Other:** Africa and Antarctica specify: _________

1. At what point in the production chain were samples taken (check all that apply)
   - Parent/ breeder flock
   - Hatchery
   - On broiler farm during first 2 weeks of broiler placement
   - On broiler farm from 2^nd^ week of placement onward
   - At processing cecal sample or cloacal swabs taken to establish *Campylobacter* status.
   - At processing after exsanguination *(exclude if this is the only sample)*
   - Other point not mentioned above, specify: ___________

**Bacteria and AMR**

1. Does the article assess antimicrobial resistance of *Campylobacter spp*. isolated?

- Yes
- No
- NA no *Campylobacter* was isolated or tested in this study.

1. Does the article describe the isolation and/or antimicrobial resistance of other bacteria? (check all that apply)

**Isolated Resistance testing**

- *Salmonella spp*.  *Salmonella spp*.:
- *E. coli*  *E. coli*
- Enterobacteriacea  Enterobacteriacea
- Other bacteria: specify ________  Other bacteria: specify ________
- No  No

1. Was antimicrobial use (AMU) reported?

- Yes, specify for what species in the study _______
- No

**If yes,** please specify the antimicrobial(s) used and details of treatment if provided by the author ____________

1. Were the laboratory methods properly described in this paper or referenced other paper?

- Yes
- No
- Referenced

Note: Yes: minimum information includes sufficient information on treatment, isolation and confirmation of *Campylobacter*. No: minimum information missing. Referenced: protocol in another paper.

1. What laboratory methods were used to isolate *Campylobacter*? (Specify briefly type of method e.g. media name or primer name)
   - Enrichment broth, specify: ______
   - Culture : specify ________

Confirmation methods:

- Cell morphology
- Gram stain
- Oxidase test
- Failure to grow in air at 25C for 48h
- Campy dry test
- Other, specify: ______
  - PCR (Polymerase Chain Reaction)
  - MLST (Multi-locus Sequence Typing)
  - PFGE (Pulsed-Field Gel Electrophoresis.)
  - CGF/CGH (Comparative Genomics Fingerprinting/Hybridization)
  - Other : specify ________
  - NA, no *Campylobacter* isolation/characterisation was done in this study

1. Was any further molecular-typing done to link *Campylobacter* isolates from different samples?
   - Yes
   - No

**Intervention:**

1. If this study examined an intervention, what was the intervention? (copy and paste or type in what intervention was investigated) _______

**Minimum Quality Criteria:**

1. Was an appropriate (define) control / comparison group selected for this study? (Note page reported or issue with the control group)
   - Yes, specify: ________
   - No, specify: ________ (this is exclusion criteria)
   - NA no control group (e.g. prevalence or cross-sectional study)

Note: appropriate control/ comparison group:

Experiment = a control group that is comparable to the treated group except for the intervention of interest.

Observational = a representative, general sampling, comparative population with/without exposure or disease of interest (depending on design) e.g. consider parent-progeny, different species appropriately sampled that are space and time relevant for comparison.

1. Was the material and methods described in sufficient detail to allow reproduction of the study?
   - - Yes
     - No
     - Referenced

Note: Yes: minimum information includes, intervention used and applied/ appropriately conducted sampling/ dosage, duration and frequency of treatment/ sampling etc.

No: minimum information missing. Referenced: protocol in another paper.

1. Is there raw or adjusted data to extract?
   - Yes : specify ________
   - No: specify ________ (Data are not reported as summary and measure of variability, numerator and denominator, or only graphical data is available. Or this is a molecular characterization study and only the results of a cluster analysis are available.)
2. Additional Comments: ________

***Appendix S1c. Risk of Bias (RoB), GRADE and data extraction***

***Vertical transfer (VT)***

| Question | Answers | Explanation |
| --- | --- | --- |
| **General Questions- VT** | | |
| Was this included in the Newell 2004 review? | □ Yes  □ No, published 2004 or earlier  □ Published since 2004 |  |
| Did the authors conclude there was evidence for vertical transmission? | □Yes  □No |  |
| Did the authors sample and report that the breeding flocks were positive? | □Yes  □No |  |
| Did the authors report the prevalence of *Campylobacter* from hatchery samples? | □Yes ________  □No *Campylobacter* isolated  □NA, did not sample hatchery |  |
| Did the authors report the prevalence of *Campylobacter* from 1 week old chicks? | □Yes ________  □No *Campylobacter* isolated  □NA, did not sample 1 week old chicks |  |
| Please specify the points of comparison/ analysis in this study. e.g., hatch only, breeders vs. 28 day broilers. |  | This was an attempt to find logical groupings of studies. |

***All studies- animals, humans, environment***

| **GRD Com** | **Study Design** | **Question** | **Answers** | **Explanation** | **Rules** |
| --- | --- | --- | --- | --- | --- |
| 1-1 | Intervention (Ct, Cht) | Was allocation sequence adequately generated? | Yes, allocation sequence is described in sufficient detail. ___page#?  Unclear - simple statement that says they "randomized"  No  NA | Selection bias, (8.5.a)  *Less of an issue with agricultural animal studies*.  Yes- random number table/ sequence generation, coin toss, etc.  No- non-random generation eg. Odd or even, date of admission, judgment of researcher etc. | No downgrade with the broiler studies for this criterion. |
| 1-2a | Intervention (Ct, Cht) | Was the allocation sequence adequately concealed from the participants and the researcher? | Yes, concealment was sufficient and allocation was unlikely to be foreseen. ___page#?  Unclear- only said they "blinding or concealed treatment"  No  NA | Selection bias (8.5.a)  *Less of an issue with agricultural animals*  Yes, central allocation, sequentially numbered drugs etc., coding of some sort.  No, if assignment is foreseen due to alternation, date of birth, case number etc.  Unclear, methods of concealment not described. | No downgrade with the broiler studies for this criterion. |
| 1-2b | Cohort | Was the level of exposure representative of exposure in the population of interest? | Yes  Unclear- too few details are available to make a clear Judgement.  No  NA | Selection bias (added)  Yes, Does the sample reflect the proportion of high risk and low risk people in the population the investigator would like to extrapolate the results to?  No, | No downgrade with the broiler studies for this criterion. |
| 1-2c | Cross-sectional & Prevalence  OR  Case Control | Were the study participants (samples) selected randomly so the sample reflects disease and exposure in the population of interest?  OR  Were the controls selected from the same source population as the cases? | Yes  Unclear- too few details are available to make a clear Judgement.  No  NA | Selection bias (added)  Yes: Random selection of the study participants or samples are stated and described.  No: Study participants were selected non-randomly or were not Described  NA | No downgrade with the broiler studies for this criterion. |
| 1-3 | All | Was blinding appropriate? (Patient, doctor, farm hand, outcome assessor, manuscript writer.) *Please note if there is a different answer for different outcomes.* | Yes,  Unclear, reported that blinding was used  No _explain_____ | Performance and detection bias. (8.5.a)  Were knowledge of the intervention/ status of the individual or sample adequately prevented during the study? | Currently no Downgrade recommended |
| 1-4 | Intervention (Ct, ChT), Cohort | Was loss to follow-up reported and equal in both groups | Yes, ___page#?___  Unclear- too few details are available to make a clear judgment.  No _explain_____  NA | Attrition Bias (8.5.a)  Yes, loss to follow-up was reported and discussed OR there was NO loss to follow-up. There is no reason to believe that the missing data is related to the outcome, balanced loss across groups, and missing data has been dealt with in the analysis.  No: there was loss to follow-up of concern (>20%), it was not clearly reported or discussed. Reason for loss is related to the study group (intervention), the as treated analysis shows there is an issue with the remaining participants.  NA - no loss to follow-up | Downgrade if there is a serious violation. |
| 1-5 | All | Were observations excluded from the analysis reported? | Yes, ___page#?  Unclear- too few details are available to make a clear judgment.  No _explain_____ | Attrition bias – (8.5.a)  Yes, all exclusions from analysis and a reasonable justification were reported OR there were NO exclusions. Thus there is no concern about this.  No, exclusions were made from the analysis and it is unclear why or what impact this has on the conclusions. There is concern about this biasing the study results. |  |
| 1-6 | All | Does the study appear to have reported all intended outcomes? | Yes, ___page#?  Unclear- too few details are available to make a clear judgment.  No _explain_____ | Reporting bias (8.5.a)  Selective reporting of results because they are statistically non-significant.  Yes, the author reported all outcomes identified in the methods and do not appear to have selectively not reported any.  No, the author did not report all outcomes identified in the methods. Outcomes are not reported in a way that they can be used in a meta-analysis. |  |
| 1-7 | All | Was the study free of other problems that could put it at a high risk of bias? | Yes  Unclear- too few details are available to make a clear judgment.  No _explain_____ | All other bias' that could put the study at risk. (8.5.a)  e.g.: non-randomization, clusters, stopping the study early without explanation, sample size intended (these are NOT more likely to have biased results)  Vs.  Obvious imbalance in baseline factors that have an influence on the outcome. Outcome assessment can become biased. Selective reporting of subgroups can be biased (these ARE more likely to have biased results)  Yes, I have no additional concerns about the design and/or conduct and reporting of this study.  No, the following are concerns I have that this study is at risk of bias. (list with page#) | Catch- all for other criteria.  Again, unless the author points something out, there is nothing to indicate here. |
| 1-8 | All | Are there any concerns that confounders have not been appropriately identified and accounted? | Yes  Raw Data  No | Confounding bias (added)  Yes: All-important confounding factors were identified, accounted for by exclusion, matching or analysis (sex, age) or are taken care of in the design of the study.  Partial: some confounders controlled but not all of them.  No: Not stated. | Down grade for serious violations. |
| 1-10 | All –  Risk of Bias | Risk of Bias Assessment based on last 11 questions | Low risk of bias  Unclear Risk of Bias  High Risk of Bias | Answers to the 8 questions above will inform the result of the RoB.  Low risk of bias, no biases was indicated in the assessment. Thus plausible bias is unlikely in all key domains (within this study). (Across studies: most studies indicate low risk)  Unclear risk of bias, there are plausible bias that raises doubt about the results as some key domains are “unclear” (within this study). (Across studies: most information is from low or unclear RoB).  High Risk of bias indicates that in one or more of the domains serious plausible bias was identified (within the study). (Across studies: The proportion of studies that are at high risk of bias is sufficient to affect the interpretation of results.) | 1) UNCLEAR RoB?  Given that the first 3 criteria are not done in poultry studies and the next three were not part of our QA, although there is the “other bias” question would capture issues not addressed in the other RoB questions. |
| 2-1 | All-  Indirectness | Does this study examine the question of interest directly? | Yes, this study directly addresses the question of interest.  No, this study indirectly examines the question of interest. | A study may indirectly address the question of interest if:  e.g., interventions we wish to compare are measured independently in two separate trials compared to controls.  e.g., the population, intervention, comparisons or outcomes were not exactly what we are trying to draw conclusions for.  * Downgrading occurs if there is reason to believe that there may be differences in the conclusions due to indirectness. | Downgraded for use of a “challenge”  No change for “normal” contamination. |
| 3-1 | All-  Heterogeneity  (May only be addressed at meta-analysis) | Does this study agree with other studies addressing the same question? If no, are there reasons why? | Yes, this study is in alignment with other studies addressing the same question.  No, measure of effect or association differs in magnitude (or direction) for the following reasons _________________ | If there is heterogeneity present for a group of studies this should be examined for an explanation e.g. population, dose, or other identified moderators. If there is NO plausible explanation, the down-grading may be recommended. | Did not downgrade for heterogeneity; have used imprecision instead to recommend downgrading. |
| 4-1 | All-  Imprecision | Consider the magnitude and precision of the results for upgrading or down grading? | Upgrade: large magnitude, precise results  No concern: results are precise  Downgrade: low power, imprecision, little confidence is the outcome measure. | Upgrade: large RR>2-RR<0.5 or vs. large RR>5-RR<0.2, reasonably precise and no threat to validity = confident in the observation of this study.  No concern: Reasonably precise, sample size was adequate. Outcome estimate is not large enough to consider upgrading.  Downgrade: Confidence intervals are wide, they include (or almost) the null even though the measure of association is not near the null, thus there is concern about the power of this study and I have low confidence in the outcome estimate. | Downgraded if the studies within the subgroup reported both protective and harmful effects.  No Change: Studies agree in direction but are not or are heterogeneous or the 95% CI crosses the line of no effect.  Upgrading (proposed): Large, homogenous overall estimate with a significant 95% CI. |
| 5-1 | All –  Publication Bias | Was this study funded by industry, was there heavy sponsor involvement? | No, There are no concerns about suppression of studies.  Yes, There was sponsor involvement. | This criteria for down-grading would be used if all or most of the trials captured are industry funded or declare heavy sponsor involvement, in which case there are concerns that studies of null or negative effect may have been suppressed from publication.  (Selective outcome reporting is covered under criteria #1.) |  |
| 6-1 | All –  Underestimation of Effect | Is there reason to believe that due to the population studied, the magnitude of effect (association) of the intervention (outcome) may be underestimated? | Yes, an underestimation is likely  No, there is no reason to believe the estimated effect is underestimated. | You would answer yes ONLY if there was good reason to think that the study underestimated the potential association or effect of an intervention due to the population that was sampled.  E.g. a drug was only tested on severely disease individuals and not on all diseased individuals, but it is likely that a better success rate would have been found if all diseased individuals were studied.  e.g. The magnitude of association was lower than it likely is in the general population because the comparison group has a similar disease which is also more likely to result in having the exposure of interest. |  |
| 7-1 | All-  Dose-response | Was a dose-response gradient detected for the intervention or exposure being examined? | Yes, dose-response gradient detected.  No, no does-response gradient reported. | If a dose response gradient is demonstrated in some or all of the studies, this increases our confidence in the findings of the study and thus we can consider upgrading the evidence. |  |
| GRADE | All intervention-outcome categories | Given the study design, answers to the above questions, what is the GRADE of this study / set of studies? | Four star scale- starting point:  **** Randomized controlled trial  ***Quasi experiment, Challenge Trial, Cohort Study, longitudinal prevalence study.  ** Challenged Quasi-experiment, Cross-sectional study, Case-control study, Prevalence study  * Case reports etc. | After a starting point is established based on study design, evaluate the individual study GRADE criteria 1-10 through 7-1 and decide if the study should be further down-graded (significant bias/ violation) or up-graded (particularly powerful, well conducted, generalizable)  When merging across studies take the GRADE of individual studies into account along with the precision and heterogeneity criteria to decide if the overall GRADE should remain, be up or down graded. | Limitations: there is some subjectivity with coming up with an overall GRADE. |

***Appendix S1e. Data extraction tool***

***Vertical Transfer***

| **Question** | **Answers** | **Explanation** |
| --- | --- | --- |
| Breeders | Individual Bird Prevalence ____  Flock prevalence _______ |  |
| Hatchery samples | Individual Bird Prevalence ____  Flock prevalence _______ |  |
| Day old Chicks | Individual Bird Prevalence ____  Flock prevalence _______ |  |
| 1 week old broilers | Individual Bird Prevalence ____  Flock prevalence _______ |  |
| 2 week old broilers | Individual Bird Prevalence ____  Flock prevalence _______ |  |
| 3 week old broilers | Individual Bird Prevalence ____  Flock prevalence _______ |  |
| 4 week old broilers | Individual Bird Prevalence ____  Flock prevalence _______ |  |
| 5 week old broilers | Individual Bird Prevalence ____  Flock prevalence _______ |  |
| 6+ week old broilers | Individual Bird Prevalence ____  Flock prevalence _______ |  |
| Results of analysis or Comparison | Outcome: _______  Comparison groups: ________  Results: ___________________ |  |
| Further information on the study e.g. conclusions |  |  |

***Other sources***

| **Question** | **Answers** | **Explanation** |
| --- | --- | --- |
| **This line represents:** |  |  |
| Sample Type: Describe the sample that this line of data represents e.g. cattle, mouse, forklift etc. |  | **What is the population being sampled 1 per line:**   - Start with broilers - Add Domestic animals - Add Wild animals - Add humans (differentiate between type farmer vs. catcher) thus risk of farmer vs. risk of visitors - Add equipment (differentiate between farm equip vs. catcher equip) - Add Inputs: Clean litter, feed, contaminated water - Add barn environment, **only presence of *Campylobacter* PRIOR to placement** (i.e.,: cleaning and disinfection processes) |
| Sample Comment: Add any important info about the sample type e.g. broiler age |  |  |
| Sample: physical or observational? |  | This is establishing whether a sample was taken for *Campylobacter* isolation or whether this sample is the result of a questionnaire looking at associations between broiler *Campylobacter* status and the presence of a risk factor |
| If physical: note whether the results being extracted are from culture, PCR or other type of isolation technique. |  |  |
| **Raw Data** |  |  |
| Unit of Observation? | Individual  flock |  |
| Positive Samples |  | Number of +ve samples for this line. |
| Total Samples |  | Total number of samples for this line. |
| **Summarized Data** |  |  |
| What does the association represent e.g., Odds of broiler flock positive. And is it adjusted by any other variable? |  |  |
| Model/Test type if applicable. |  | Note if this the result of a logistic regression model, M-H odds etc. |
| What is the effect estimate category, please specify OR, RR etc. and measure of variation e.g. SE or 95% CI etc. | OR, SE  OR, 95%CI  RR, SE |  |
| Effect Estimate |  | Specify the reported value of the OR, RR or whatever is reported by the author. |
| Measure of variability |  | Specify the reported value of the SE, 95% CI etc. that is reported by the author. |
| *p*-value / significance? |  | Please record the *p*-value and/ or the significance of this association. |
| **Molecular Epidemiology Conclusions** |  |  |
| Did the author find a molecular link between the *Campylobacter* isolated in broilers and this sample? | Yes, an exact match  Possible, closely related but not exact  No, unlikely to be related  Not tested | This is a difficult question that is not consistently reported in the literature; if reported, authors used different terminologies. The term “flock-matched” will be used in this review to describe broiler isolates that are identical to the different sources examined. |
| Comments about the molecular epidemiology methods used |  | Include other parameters such as analytical and mathematical models. |
| Indicate other comments/important notes from the paper |  | This is an open column(s), feel free to put discussion points in here. |
