# Supplementary material for: A Systematic Review Characterizing On-Farm Sources of Campylobacter spp. for Broiler Chickens
Source: PLoS One. 2014 Aug 29;9(8):e104905. doi: 10.1371/journal.pone.0104905 (PMC4149356; doi:10.1371/journal.pone.0104905)
Supplement: Appendix S2 — List of the 95 references used in the study. (DOCX) [file pone.0104905.s003.docx]

**Appendix S2. List of the 95 references**

1. Allen VM, Ridley AM, Harris JA, Newell DG, Powell L (2011) Influence of production system on the rate of onset of *Campylobacter* colonization in chicken flocks reared extensively in the United Kingdom. Br Poult Sci 52: 30-39.

2. Allen VM, Weaver H, Ridley AM, Harris JA, Sharma M, et al. (2008) Sources and spread of thermophilic *Campylobacter* spp. during partial depopulation of broiler chicken flocks. J Food Prot 71: 264-270.

3. Ansari-Lari M, Hosseinzadeh S, Shekarforoush SS, Abdollahi M, Berizi E (2011) Prevalence and risk factors associated with *Campylobacter* infections in broiler flocks in Shiraz, southern Iran. Int J Food Microbiol 144: 475-479.

4. Bates C, Hiett KL, Stern NJ (2004) Relationship of *Campylobacter* isolated from poultry and from darkling beetles in New Zealand. Avian Dis 48: 138-147.

5. Berndtson E, Emanuelson U, Engvall A, Danielsson-Tham ML (1996) A 1-year epidemiological study of *Campylobacter*s in 18 Swedish chicken farms. Prev Vet Med 26: 167-185.

6. Berndtson E, Danielsson-Tham ML, Engvall A (1996) *Campylobacter* incidence on a chicken farm and the spread of *Campylobacter* during the slaughter process. Int J Food Microbiol 32: 35-47.

7. Bouwknegt M, van de Giessen AW, Dam-Deisz WD, Havelaar AH, Nagelkerke NJ, et al. (2004) Risk factors for the presence of *Campylobacter* spp. in Dutch broiler flocks. Prev Vet Med 62: 35-49.

8. Bull SA, Allen VM, Domingue G, Jorgensen F, Frost JA, et al. (2006) Sources of *Campylobacter* spp. colonizing housed broiler flocks during rearing. Appl Environ Microbiol 72: 645-652.

9. Byrd J, Bailey RH, Wills R, Nisbet D (2007) Recovery of *Campylobacter* from commercial broiler hatchery trayliners. Poult Sci 86: 26-29.

10. Cardinale E, Tall F, Gueye EF, Cisse M, Salvat G (2004) Risk factors for *Campylobacter* spp. infection in Senegalese broiler-chicken flocks. Prev Vet Med 64: 15-25.

11. Chuma T, Yamada T, Yano K, Okamoto K, Yugi H (1994) A survey of *Campylobacter* *coli* in broilers from assignment to slaughter using DNA-DNA hybridization. J Vet Med Sci 56: 697-700.

12. Chuma T, Makino K, Okamoto K, Yugi H (1997) Analysis of distribution of *Campylobacter* *jejuni* and *Campylobacter* *coli* in broilers by using restriction fragment length polymorphism of flagellin gene. J Vet Med Sci 59: 1011-1015.

13. Chuma T, Yano K, Omori H, Okamoto K, Yugi H (1997) Direct detection of *Campylobacter* *jejuni* in chicken cecal contents by PCR. J Vet Med Sci 59: 85-87.

14. Cokal Y (2011) The presence of *Campylobacter* *jejuni* in broiler houses: Results of a longitudinal study. African J Microbiol Res 5: 389-393.

15. Colles FM, Dingle KE, Cody AJ, Maiden MC (2008) Comparison of *Campylobacter* populations in wild geese with those in starlings and free-range poultry on the same farm. Appl Environ Microbiol 74: 3583-3590.

16. Cox NA, Stern NJ, Hiett KL, Berrang ME (2002) Identification of a new source of *Campylobacter* contamination in poultry: Transmission from breeder hens to broiler chickens. Avian Dis 46: 535-541.

17. Cox NA, Stern NJ, Musgrove MT, Bailey JS, Craven SE, et al. (2002) Prevalence and level of *Campylobacter* in commercial broiler breeders (parents) and broilers. J Appl Poult Res 11: 187-190.

18. Damjanova I, Jakab M, Farkas T, Meszaros J, Galantai Z, et al. (2011) From farm to fork follow-up of thermotolerant *Campylobacter*s throughout the broiler production chain and in human cases in a Hungarian county during a ten-months period. Int J Food Microbiol 150: 95-102.

19. Ellis-Iversen J, Jorgensen F, Bull S, Powell L, Cook AJ, et al. (2009) Risk factors for *Campylobacter* colonisation during rearing of broiler flocks in Great Britain. Prev Vet Med 89: 178-184.

20. Ellis-Iversen J, Ridley A, Morris V, Sowa A, Harris J, et al. (2011) Persistent environmental reservoirs on farms as risk factors for *Campylobacter* in commercial poultry. Epidemiol Infect : 1-9. doi:10.1017/S095026881100118X.

21. Fonseca BB, Soncini RA, Rodrigues Gimaraes A, Aparecida Rossi D (2006) *Campylobacter* spp. in eggs from cloacal swab positive breeder hens. Brazilian J Microbiol 37: 573-575.

22. Gibbens JC, Pascoe SJ, Evans SJ, Davies RH, Sayers AR (2001) A trial of biosecurity as a means to control *Campylobacter* infection of broiler chickens. Prev Vet Med 48: 85-99.

23. Giessen Avd, Mazurier SI, Jacobs-Reitsma W, Jansen W, Berkers P, et al. (1992) Study on the epidemiology and control of *Campylobacter* *jejuni* in poultry broiler flocks. Appl Environ Microbiol 58: 1913-1917.

24. Giessen AWvd, Bloemberg BPM, Ritmeester WS, Tilburg JJHC (1996) Epidemiological study on risk factors and risk reducing measures for *Campylobacter* infections in Dutch broiler flocks. Epidemiol Infect 117: 245-250.

25. van de Giessen AW, Tilburg JJ, Ritmeester WS, van der Plas J (1998) Reduction of *Campylobacter* infections in broiler flocks by application of hygiene measures. Epidemiol Infect 121: 57-66.

26. Gregory E, Barnhart H, Dreesen D, Stern N, Corn J (1997) Epidemiological study of *Campylobacter* spp. in broilers: Source, time of colonization, and prevalence. Avian Dis 41: 890-898.

27. Guerin MT, Martin W, Reiersen J, Berke O, McEwen SA, et al. (2007) A farm-level study of risk factors associated with the colonization of broiler flocks with *Campylobacter* spp. in Iceland, 2001-2004. Acta Vet Scand 49: 18.

28. Hald B, Skovgard H, Bang DD, Pedersen K, Dybdahl J, et al. (2004) Flies and *Campylobacter* infection of broiler flocks. Emerg Infect Dis 10: 1490-1492.

29. Hald B, Sommer HM, Skovgard H (2007) Use of fly screens to reduce *Campylobacter* spp. introduction in broiler houses. Emerg Infect Dis 13: 1951-1953.

30. Hald B, Wedderkopp A, Madsen M (2000) Thermophilic *Campylobacter* spp. in Danish broiler production: A cross-sectional survey and a retrospective analysis of risk factors for occurrence in broiler flocks. Avian Pathol 29: 123-131.

31. Hald B, Skovgård H, Pedersen K, Bunkenborg H (2008) Influxed insects as vectors for *Campylobacter* *jejuni* and *Campylobacter* *coli* in Danish broiler houses. Poult Sci 87: 1428-1434.

32. Herman L, Heyndrickx M, Grijspeerdt K, Vandekerchove D, Rollier I, et al. (2003) Routes for *Campylobacter* contamination of poultry meat: Epidemiological study from hatchery to slaughterhouse. Epidemiol Infect 131: 1169-1180.

33. Hansson I, Ederoth M, Andersson L, Vagsholm I, Engvall E (2005) Transmission of *Campylobacter* spp. to chickens during transport to slaughter. J Appl Microbiol 99: 1149-1157.

34. Hansson I, Vagsholm I, Svensson L, Engvall EO (2007) Correlations between *Campylobacter* spp. prevalence in the environment and broiler flocks. J Appl Microbiol 103: 640-649.

35. Hansson I, Engvall EO, Vagsholm I, Nyman A (2010) Risk factors associated with the presence of *Campylobacter*-positive broiler flocks in Sweden. Prev Vet Med 96: 114-121.

36. Hiett KL, Cox NA, Stern NJ (2002) Direct polymerase chain reaction detection of *Campylobacter* spp. in poultry hatchery samples. Avian Dis 46: 219-223.

37. Hiett K, Stern N, Fedorka-Cray P, Cox N, Musgrove M, et al. (2002) Molecular subtype analyses of *Campylobacter* spp. from Arkansas and California poultry operations. Appl Environ Microbiol 68: 6220-6236.

38. Hiett KL, Stern NJ, Fedorka-Cray P, Cox NA, Seal BS (2007) Molecular phylogeny of the *fla*A short variable region among *Campylobacter* *jejuni* isolates collected during an annual evaluation of poultry flocks in the southeastern United States. Foodborne Pathog Dis 4: 339-347.

39. Humphrey TJ, Henley A, Lanning DG (1993) The colonization of broiler chickens with *Campylobacter* *jejuni*: Some epidemiological investigations. Epidemiol Infect 110: 601-607.

40. Huneau-Salaun A, Denis M, Balaine L, Salvat G (2007) Risk factors for *Campylobacter* spp. colonization in french free-range broiler-chicken flocks at the end of the indoor rearing period. Prev Vet Med 80: 34-48.

41. Idris U, Lu J, Maier M, Sanchez S, Hofacre CL, et al. (2006) Dissemination of fluoroquinolone-resistant *Campylobacter* spp. within an integrated commercial poultry production system. Appl Environ Microbiol 72: 3441-3447.

42. Jacobs-Reitsma WF, van de Giessen AW, Bolder NM, Mulder RW (1995) Epidemiology of *Campylobacter* spp. at two Dutch broiler farms. Epidemiol Infect 114: 413-421.

43. Johnsen G, Kruse H, Hofshagen M (2006) Genetic diversity and description of transmission routes for *Campylobacter* on broiler farms by amplified-fragment length polymorphism. J Appl Microbiol 101: 1130-1139.

44. Jones FT, Axtell SP, Rives DV, Scheideler FR, Tarver JR, et al. (1991) A survey of *Campylobacter* *jejuni* contamination in modern broiler production and processing plant. J Food Protection 54: 259-262.

45. Jozwiak A, Reichart O, Laczay P (2006) The occurrence of *Campylobacter* species in Hungarian broiler chickens from farm to slaughter. J Vet Med Series B-Infectious Diseases and Veterinary Public Health 53: 291-294.

46. Kapperud G, Skjerve E, Vik L, Hauge K, Lysaker A, et al. (1993) Epidemiological investigation of risk factors for *Campylobacter* colonization in Norwegian broiler flocks. Epidemiol Infect 111: 245-255.

47. Kassem II, Sanad Y, Gangaiah D, Lilburn M, Lejeune J, et al. (2010) Use of bioluminescence imaging to monitor *Campylobacter* survival in chicken litter. J Appl Microbiol 109: 1988-1997.

48. Kazwala RR, Collins JD, Hannan J (1992) The establishment and spread of experimental *Campylobacter* *jejuni* infections in young chickens. Prev Vet Med 13: 19-26.

49. Kazwala RR, Collins JD, Hannan J, Crinion RA, O'Mahony H (1990) Factors responsible for the introduction and spread of *Campylobacter* *jejuni* infection in commercial poultry production. Vet Rec 126: 305-306.

50. Kuana SL, Santos LR, Rodriquez LB, Borsoi A, Moraes HLS, et al. (2007) Risk factors and likelihood of *Campylobacter* colonization in broiler flocks. Brazilian J Poultry Sci 9: 201-204.

51. Lyngstad TM, Jonsson ME, Hofshagen M, Heier BT (2008) Risk factors associated with the presence of *Campylobacter* species in Norwegian broiler flocks. Poult Sci 87: 1987-1994.

52. McCrea BA, Macklin KS, Norton RA, Hess JB, Bilgili SF (2006) A longitudinal study of Salmonella and *Campylobacter* *jejuni* isolates from day of hatch through processing by automated ribotyping. J Food Prot 69: 2908-2914.

53. McDowell SW, Menzies FD, McBride SH, Oza AN, McKenna JP, et al. (2008) *Campylobacter* spp. in conventional broiler flocks in Northern Ireland: Epidemiology and risk factors. Prev Vet Med 84: 261-276.

54. Messens W, Herman L, De Zutter L, Heyndrickx M (2009) Multiple typing for the epidemiological study of contamination of broilers with thermotolerant *Campylobacter*. Vet Microbiol 138: 120-131.

55. Nather G, Alter T, Martin A, Ellerbroek L (2009) Analysis of risk factors for *Campylobacter* species infection in broiler flocks. Poult Sci 88: 1299-1305.

56. Nesbit EG, Gibbs P, Dreesen DW, Lee MD (2001) Epidemiologic features of *Campylobacter* *jejuni* isolated from poultry broiler houses and surrounding environments as determined by use of molecular strain typing. Am J Vet Res 62: 190-194.

57. Neubauer C, Bibl D, Szolgyenyi W, Jauk V, Schmidt M, et al. (2005) Epidemiological investigation of *Campylobacter* spp. in Austrian broiler flocks: Prevalence and risk factors. Ved Med Austria 92: 4-10.

58. Ogden ID, MacRae M, Johnston M, Strachan NJ, Cody AJ, et al. (2007) Use of multilocus sequence typing to investigate the association between the presence of *Campylobacter* spp. in broiler drinking water and *Campylobacter* colonization in broilers. Appl Environ Microbiol 73: 5125-5129.

59. Olsen KN, Lund M, Skov J, Christensen LS, Hoorfar J (2009) Detection of *Campylobacter* bacteria in air samples for continuous real-time monitoring of *Campylobacter* colonization in broiler flocks. Appl Environ Microbiol 75: 2074-2078.

60. O'Mahony E, Buckley JF, Bolton D, Whyte P, Fanning S (2011) Molecular epidemiology of *Campylobacter* isolates from poultry production units in southern ireland. PLoS One 6: e28490. Available: <http://www.plosone.org/article/fetchObject.action?uri=info%3Adoi%2F10.1371%2Fjournal.pone.0028490&representation=PDF>. Accessed 21 July 2014.

61. Patriarchi A, Fox A, Maunsell B, Fanning S, Bolton D (2011) Molecular characterization and environmental mapping of *Campylobacter* isolates in a subset of intensive poultry flocks in Ireland. Foodborne Pathog Dis 8: 99-108.

62. Patriarchi A, Maunsell B, O'Mahony E, Fox A, Fanning S, et al. (2009) Prevalence of *Campylobacter* spp. in a subset of intensive poultry flocks in Ireland. Lett Appl Microbiol 49: 305-310.

63. Payne RE, Lee MD, Dreesen DW, Barnhart HM (1999) Molecular epidemiology of *Campylobacter* *jejuni* in broiler flocks using randomly amplified polymorphic DNA-PCR and 23S rRNA-PCR and role of litter in its transmission. Appl Environ Microbiol 65: 260-263.

64. Pearson AD, Greenwood M, Healing TD, Rollins D, Shahamat M, et al. (1993) Colonization of broiler chickens by waterborne *Campylobacter* *jejuni*. Appl Environ Microbiol 59: 987-996.

65. Pearson AD, Greenwood MH, Feltham RK, Healing TD, Donaldson J, et al. (1996) Microbial ecology of *Campylobacter* *jejuni* in a United Kingdom chicken supply chain: Intermittent common source, vertical transmission, and amplification by flock propagation. Appl Environ Microbiol 62: 4614-4620.

66. Petersen L, Nielsen EM, On SL. (2001) Serotype and genotype diversity and hatchery transmission of *Campylobacter* *jejuni* in commercial poultry flocks. Vet Microbiol 82: 141-154.

67. Pokamunski S, Kass N, Borochovich E, Marantz B, Rogol M (1986) Incidence of *Campylobacter* spp. in broiler flocks monitored from hatching to slaughter. Avian Pathol 15: 83-92.

68. Ramabu SS, Boxall NS, Madie P, Fenwick SG (2004) Some potential sources for transmission of *Campylobacter* *jejuni* to broiler chickens. Lett Appl Microbiol 39: 252-256.

69. Rasschaert G, Houf K, De Zutter L (2007) External contamination of *Campylobacter*-free flocks after transport in cleaned and disinfected containers. J Food Prot 70: 40-46.

70. Refregier-Petton J, Rose N, Denis M, Salvat G. (2001) Risk factors for *Campylobacter* spp. contamination in french broiler-chicken flocks at the end of the rearing period. Prev Vet Med 50: 89-100.

71. Ridley A, Morris V, Gittins J, Cawthraw S, Harris J, et al. (2011) Potential sources of *Campylobacter* infection on chicken farms: Contamination and control of broiler-harvesting equipment, vehicles and personnel. J Appl Microbiol 111: 233-244.

72. Ridley AM, Allen VM, Sharma M, Harris JA, Newell DG (2008) Real-time PCR approach for detection of environmental sources of *Campylobacter* strains colonizing broiler flocks. Appl Environ Microbiol 74: 2492-2504.

73. Ridley AM, Morris VK, Cawthraw SA, Ellis-Iversen J, Harris JA, et al. (2011) Longitudinal molecular epidemiological study of thermophilic *Campylobacter*s on one conventional broiler chicken farm. Appl Environ Microbiol 77: 98-107.

74. Rivoal K, Ragimbeau C, Salvat G, Colin P, Ermel G (2005) Genomic diversity of *Campylobacter* *coli* and *Campylobacter* *jejuni* isolates recovered from free-range broiler farms and comparison with isolates of various origins. Appl Environ Microbiol 71: 6216-6227.

75. Rodenburg TB, Van Der Hulst-Van Arkel, M.C., Kwakkel RP (2004) *Campylobacter* and *Salmonella* infections on organic broiler farms. NJAS 52: 101-108.

76. Rosef O, Kapperud G (1983) House flies (Musca domestica) as possible vectors of *Campylobacter* *fetus* subsp. *jejuni*. Appl Environ Microbiol 45: 381-383.

77. Rushton SP, Humphrey TJ, Shirley MD, Bull S, Jorgensen F (2009) *Campylobacter* in housed broiler chickens: A longitudinal study of risk factors. Epidemiol Infect 137: 1099-1110.

78. Russa AD, Bouma A, Vernooij JC, Jacobs-Reitsma W, Stegeman JA (2005) No association between partial depopulation and *Campylobacter* spp. colonization of Dutch broiler flocks. Lett Appl Microbiol 41: 280-285.

79. Sahin O, Kobalka P, Zhang Q (2003) Detection and survival of *Campylobacter* in chicken eggs. J Appl Microbiol 95: 1070-1079.

80. Sasaki Y, Tsujiyama Y, Tanaka H, Yoshida S, Goshima T, et al. (2011) Risk factors for *Campylobacter* colonization in broiler flocks in Japan. Zoonoses Public Health 58: 350-356.

81. Shane SM, Montrose MS, Harrington KS (1985) Transmission of *Campylobacter* *jejuni* by the housefly (Musca domestica). Avian Dis 29: 384-391.

82. Shanker S, Lee A, Sorrell TC (1990) Horizontal transmission of *Campylobacter* *jejuni* amongst broiler chicks: Experimental studies. Epidemiol Infect 104: 101-110.

83. Shanker S, Lee A, Sorrell TC (1986) *Campylobacter* *jejuni* in broilers: The role of vertical transmission. J Hyg (Lond) 96: 153-159.

84. Shreeve JE, Toszeghy M, Ridley A, Newell DG (2002) The carry-over of *Campylobacter* isolates between sequential poultry flocks. Avian Dis 46: 378-385.

85. Skov MN, Spencer AG, Hald B, Petersen L, Nauerby B, et al. (2004) The role of litter beetles as potential reservoir for *Salmonella enterica* and thermophilic *Campylobacter* spp. between broiler flocks. Avian Dis 48: 9-18.

86. Slader J, Domingue G, Jorgensen F, McAlpine K, Owen RJ, et al. (2002) Impact of transport crate reuse and of catching and processing on *Campylobacter* and *Salmonella* contamination of broiler chickens. Appl Environ Microbiol 68: 713-719.

87. Snelling WJ, McKenna JP, Lecky DM, Dooley JS (2005) Survival of *Campylobacter* *jejuni* in waterborne protozoa. Appl Environ Microbiol 71: 5560-5571.

88. Stern NJ, Fedorka-Cray P, Bailey JS, Cox NA, Craven SE, et al. (2001) Distribution of *Campylobacter* spp. in selected U.S. poultry production and processing operations. J Food Prot 64: 1705-1710.

89. Stern NJ, Robach MC, Cox NA, Musgrove MT (2002) Effect of drinking water chlorination on *Campylobacter* spp. colonization of broilers. Avian Dis 46: 401-404.

90. Studer E, Luthy J, Hubner P (1999) Study of the presence of *Campylobacter* *jejuni* and *C. coli* in sand samples from four Swiss chicken farms. Res Microbiol 150: 213-219.

91. Templeton JM, De Jong AJ, Blackall PJ, Miflin JK (2006) Survival of *Campylobacter* spp. in darkling beetles (*Alphitobius diaperinus*) and their larvae in Australia. Appl Environ Microbiol 72: 7909-7911.

92. Vandeplas S (2010) Prevalence and sources of *Campylobacter* spp. contamination in free-range broiler production in the southern part of Belgium. Biotechnol Agron Soc Environ 14: 279-288.

93. Workman SN, Mathison GE, Lavoie MC (2008) An investigation of sources of *Campylobacter* in a poultry production and packing operation in Barbados. Int J Food Microbiol 121: 106-111.

94. Zimmer M, Barnhart H, Idris U, Lee MD (2003) Detection of *Campylobacter* *jejuni* strains in the water lines of a commercial broiler house and their relationship to the strains that colonized the chickens. Avian Dis 47: 101-107.

95. Zweifel C, Scheu KD, Keel M, Renggli F, Stephan R (2008) Occurrence and genotypes of *Campylobacter* in broiler flocks, other farm animals, and the environment during several rearing periods on selected poultry farms. Int J Food Microbiol 125: 182-187.
